# Epigenetic regulators Rbbp4 and Hdac1 are overexpressed in a zebrafish model of RB1 embryonal brain tumor, and are required for neural progenitor survival and proliferation

**DOI:** 10.1242/dmm.034124

**Published:** 2018-06-15

**Authors:** Laura E. Schultz, Jeffrey A. Haltom, Maira P. Almeida, Wesley A. Wierson, Staci L. Solin, Trevor J. Weiss, Jordan A. Helmer, Elizabeth J. Sandquist, Heather R. Shive, Maura McGrail

**Affiliations:** 1 Department of Genetics, Development and Cell Biology, Iowa State University, Ames, IA 50011, USA; 2 Department of Population Health and Pathobiology, North Carolina State University, Raleigh, NC 27607, USA

**Keywords:** Zebrafish, Rbbp4, Hdac1, RB1 embryonal oligoneural brain tumor, Epigenetic regulators, CRISPR somatic targeting, Neural progenitor

## Abstract

In this study, we used comparative genomics and developmental genetics to identify epigenetic regulators driving oncogenesis in a zebrafish *retinoblastoma 1* (*rb1*) somatic-targeting model of RB1 mutant embryonal brain tumors. Zebrafish *rb1* brain tumors caused by TALEN or CRISPR targeting are histologically similar to human central nervous system primitive neuroectodermal tumors (CNS-PNETs). Like the human oligoneural *OLIG2+/SOX10+* CNS-PNET subtype, zebrafish *rb1* tumors show elevated expression of neural progenitor transcription factors *olig2*, *sox10*, *sox8b* and the receptor tyrosine kinase *erbb3a* oncogene. Comparison of *rb1* tumor and *rb1/rb1* germline mutant larval transcriptomes shows that the altered oligoneural precursor signature is specific to tumor tissue**.** More than 170 chromatin regulators were differentially expressed in *rb1* tumors, including overexpression of chromatin remodeler components *histone deacetylase 1* (*hdac1*) and *retinoblastoma binding protein 4* (*rbbp4*). Germline mutant analysis confirms that zebrafish *rb1*, *rbbp4* and *hdac1* are required during brain development. *rb1* is necessary for neural precursor cell cycle exit and terminal differentiation, *rbbp4* is required for survival of postmitotic precursors, and *hdac1* maintains proliferation of the neural stem cell/progenitor pool. We present an *in vivo* assay using somatic CRISPR targeting plus live imaging of histone-H2A.F/Z-GFP fusion protein in developing larval brain to rapidly test the role of chromatin remodelers in neural stem and progenitor cells. Our somatic assay recapitulates germline mutant phenotypes and reveals a dynamic view of their roles in neural cell populations. Our study provides new insight into the epigenetic processes that might drive pathogenesis in RB1 brain tumors, and identifies Rbbp4 and its associated chromatin remodeling complexes as potential target pathways to induce apoptosis in RB1 mutant brain cancer cells.

This article has an associated First Person interview with the first author of the paper.

## Abstract (precis)

**Summary:** This study shows that chromatin remodelers that are overexpressed in a zebrafish model of RB1 mutant brain cancer are required for neural progenitor proliferation and survival, providing insight into potential mechanisms that drive tumor growth.

## INTRODUCTION

The retinoblastoma tumor suppressor Retinoblastoma 1 (RB1) plays distinct roles in regulating proliferation and differentiation in stem, progenitor and lineage-restricted cell populations (Fong and Slack, 2017; Julian and Blais, 2015; Sage, 2012). The canonical tumor suppressor role of RB1 is to regulate proliferation by transcriptional repression of E2F targets driving cell cycle entry (Dyson, 2016). RB1 might also function as a tumor suppressor by promoting differentiation of lineage-committed cells (Calo et al., 2010) and survival of postmitotic neurons (Naser et al., 2016; Vandenbosch et al., 2016), and preventing cellular reprogramming (Kareta et al., 2015). RB1 transcriptional control is mediated, in part, through association with chromatin remodelers that modify post-translational marks on histones to activate or repress gene expression (Beshiri et al., 2012; Brehm et al., 1998; Lin et al., 2011). Like RB1, many chromatin remodelers are mutated in human cancers (Vogelstein et al., 2013), implicating epigenetic control of gene expression as a significant contributor to oncogenesis (Conway et al., 2015; Suva et al., 2013). Recent analyses of the epigenome in human and mouse retinoblastoma show that histone modification correlates with oncogenic gene expression (Aldiri et al., 2017; Benavente et al., 2013; Zhang et al., 2012). Understanding how RB1 loss affects epigenetic control in neural stem and progenitor cells during development and in brain cancer is important for identifying new pathways that contribute to carcinogenesis.

Two epigenetic regulators that directly interact with RB1 are the retinoblastoma binding protein RBBP4 (Qian and Lee, 1995; Qian et al., 1993) and the Class I histone deacetylase HDAC1 (Brehm et al., 1998; Luo et al., 1998; Magnaghi-Jaulin et al., 1998). RBBP4 is a WD40-repeat histone chaperone (Burkhart and Sage, 2008; Murzina et al., 2008; Nowak et al., 2011; Song et al., 2008) and a chromatin adaptor component of multiple remodeling complexes, including the nucleosome remodeling and histone deacetylase complex (NuRD) (Alqarni et al., 2014) and the cell cycle regulatory DREAM complex (Harrison et al., 2006; Litovchick et al., 2007). RBBP4 expression is altered in human glioma (Forbes et al., 2017), and was recently shown to cooperate with the p300 (EP300) histone acetyl transferase to activate DNA repair pathway gene expression in glioblastoma cells in response to temozolomide (Kitange et al., 2016). HDAC1 is a component of NuRD and other repressive complexes which control maintenance of stem cell pluripotency and cell differentiation (Hayakawa and Nakayama, 2011). Studies in zebrafish show that *hdac1* is necessary for central and peripheral nervous system development (Henion et al., 1996; Ignatius et al., 2013), and is required for cell cycle exit and differentiation of neural precursors in the retina (Stadler et al., 2005; Yamaguchi et al., 2005). The role of HDAC1 in promoting proliferation versus differentiation could depend on the type and location of the neural cell population examined (Jaworska et al., 2015). Examining the contribution of HDAC1 and RBBP4 to maintaining the progenitor-like state of RB1 brain tumors would shed light on the mechanism of chromatin remodeling in epigenetic control of tumor suppression.

We previously demonstrated that genome editing nucleases can be used to model brain tumors in zebrafish by targeted somatic inactivation of the *rb1* tumor suppressor gene (Solin et al., 2015). Transcription activator-like effector nuclease (TALEN) targeting of zebrafish *rb1* leads to brain tumors with histological similarity to central nervous system primitive neuroectodermal tumors (CNS-PNETs) (Solin et al., 2015). The PNETs are a group of aggressive, poorly differentiated tumors that feature neuroblast-like cells, which suggests that this class of tumor originates from a progenitor population that mirrors the embryonic neuroectoderm (Ostrom et al., 2017; Chan et al., 2015). Recently, other zebrafish embryonal PNET models have been created by somatic targeting or oncogene overexpression. Targeting *rb1* in a *tp53* mutant background produces medulloblastoma-like PNETs arising in the zebrafish hindbrain (Shim et al., 2017). Activation of RAS/MAPK signaling by *NRAS* overexpression in zebrafish oligoneural precursors leads to PNETs (Modzelewska et al., 2016) that molecularly resemble the human oligoneural PNET subtype, *OLIG2+/SOX10+* CNS-PNET (Picard et al., 2012; Sturm et al., 2016), defined by elevated expression of the neural progenitor transcription factors OLIG2, SOX10, SOX8 and SOX2. Together, these models suggest that disruption of multiple cellular pathways can lead to the formation of PNETs. Whether epigenetic mechanisms also contribute to zebrafish embryonal PNET oncogenesis, as suggested by genomic analyses of human and mouse tumors, remains to be examined.

Here, we use transcriptomics, somatic and germline CRISPR/Cas9 mutagenesis, and live-cell imaging in zebrafish to identify candidate RB1-interacting chromatin remodelers and examine their role in neural stem and progenitor cells during development. Our analyses provide new insight into the genomic processes that drive oncogenesis in RB1 mutant brain tumors. Comparative transcriptome analysis of zebrafish *rb1* brain tumors with *rb1/rb1* homozygous mutant tissue suggests elevated expression of oligoneural precursor transcription factors, and chromatin remodelers distinguish neoplastic from mutant tissue. Isolation of *rb1* germline mutants shows that in the developing nervous system, *rb1* is required cell autonomously to block cell cycle re-entry in neural precursors. We demonstrate that the chromatin remodeling adaptor and histone chaperone *rbbp4* is necessary for the survival of neural precursors, and that in the absence of *rbbp4*, neural precursor cells undergo apoptosis. In contrast, *hdac1* is necessary to maintain proliferation in neural stem/progenitor cells. CRISPR somatic targeting recapitulates germline mutagenesis phenotypes. Live-cell imaging of histone H2A-GFP in mutant larvae reveals a dynamic view of the effect of gene loss on neural stem and progenitor cell division and survival. *rb1* mutant neural precursors re-enter the cell cycle but appear to stall in early mitosis, indicating a requirement for zebrafish Rb1 in initiating quiescence as well as progression through the cell cycle. Our genomic and phenotypic comparisons indicate that in *rb1* neoplastic cells, elevated levels of *rbbp4* and *hdac1* might contribute to proliferation and survival. Together, these results identify Rbbp4 and its associated chromatin remodelers as a potential new target for inhibiting tumor cell survival by driving neural cancer stem cells into apoptosis.

## RESULTS

### Zebrafish *rb1* brain tumors model embryonal *OLIG2+/SOX10+* CNS-PNETs

**Fig. 1.**
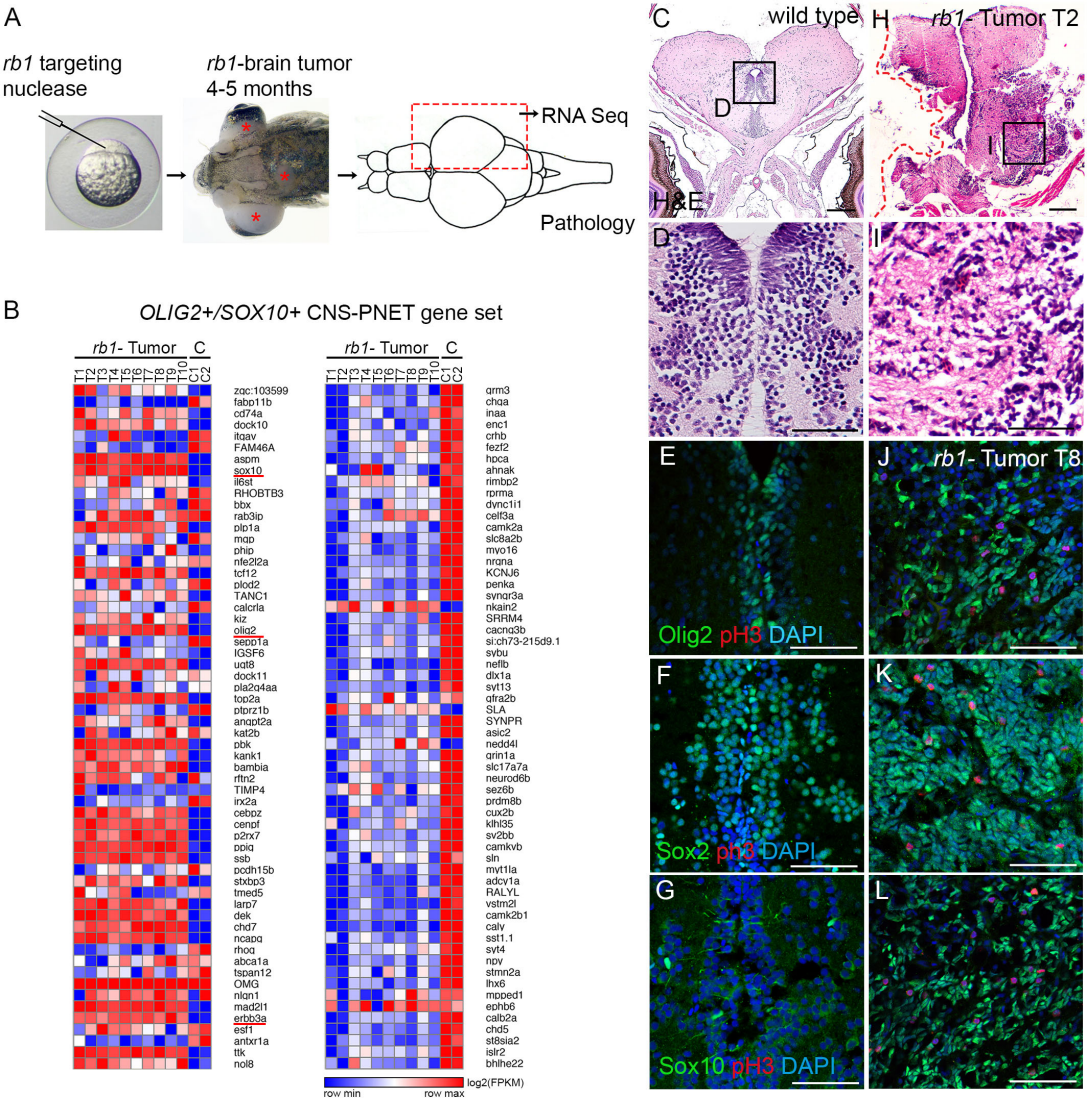
**Zebrafish *rb1* brain tumors express an oligoneural precursor signature similar to human *OLIG2+/SOX10+* CNS-PNET.** (A) Zebrafish *rb1* brain tumor model created by somatic targeting of *rb1* exon 2 with CRISPR-Cas9 or TALENs. Gross brain tumors develop in the midbrain and hindbrain (red asterisks) of adults at 4-5 months of age. For transcriptome analysis, half of the tumor tissue was dissected from ten tumor-positive fish for RNA isolation. The remaining tissue was embedded for pathology and immunolabeling. (B) Heat maps of log2(FPKM) show differential gene expression in zebrafish *rb1* brain tumor transcriptome of 120 genes in the human *OLIG2+/SOX10+* CNS-PNET subtype. *rb1* tumor RNA-Seq of ten biological replicates from ten individual tumor positive adults, and C RNA-Seq of two biological replicates of three pooled normal adult zebrafish brain, are shown. (C-L) Histological and immunolabeling of wild-type adult zebrafish pretectum/diencephalon (C-G) and tumor-containing brain tissue from transcriptome individual T2 (H,I) and T8 (J-L). Neoplastic cells in T2 tumor show small densely basophilic nuclei, consistent with primitive neuroectodermal-like tumors (H,I). In normal adult brain, Olig2, Sox2 and Sox10 labeling is restricted to cells at or adjacent to the ventricle (E-G). High levels of Olig2, Sox2 and Sox10 are detected in the neoplastic tissue in tumor T8. Phosphohistone H3-labeled mitotic cells are scattered throughout the lesion (J-L). Scale bars: 200 µm (C,H); 50 µm (E,F,G,J,K,L).

We previously developed a zebrafish *rb1* brain tumor model that resembles poorly differentiated primitive neuroectodermal tumors by somatic targeting of zebrafish *rb1* with TALENs (Solin et al., 2015). Targeting produces mosaic adults that develop brain tumors at 4-5 months of age (Fig. 1A). To determine the molecular signature of the zebrafish *rb1* brain tumors, ten tumor-bearing adults were dissected. Half of the tumor tissue was used for RNA isolation, and half was embedded for histological analysis (Fig. 1A; Fig. S1). RNA sequencing (RNA-Seq) libraries were prepared from the ten tumor biological replicates and two pools of three normal adult brains (Fig. S2) and analyzed for differentially expressed genes (Tables S1-S3). E2F targets driving cell cycle entry were highly upregulated, and Ingenuity Pathway Analysis (IPA) revealed activation of mitosis, cell cycle, and DNA replication, recombination and repair pathways (Fig. S3). Gene Set Enrichment Analysis (GSEA) also indicated a positive correlation with RB1-E2F oncogenic cell signaling and transcriptional regulation pathways, and a negative correlation with neurological differentiation (Fig. S4). These results are consistent with misregulation of RB1-dependent pathways driving oncogenesis in the *rb1* brain tumors.

We next compared the zebrafish *rb1* brain tumor transcriptome to other zebrafish brain tumor models and to human CNS-PNET. A set of 120 genes most highly up- and downregulated in the human *OLIG2+/SOX10+* CNS-PNET subtype (Picard et al., 2012) was previously used to analyze differential gene expression in a zebrafish *NRAS* CNS-PNET model (Modzelewska et al., 2016). The zebrafish *rb1* brain tumors showed a similar pattern of differential gene expression across the 60 up- and 60 downregulated genes. Defining factors of the human oligoneural CNS-PNET subgroup, *olig2*, *sox10*, *sox8b* and *erbb3a*, and the stem/progenitor marker *sox2* (Fig. 1B; Tables S2 and S3) were upregulated in the zebrafish *rb1* tumors. Histopathological analyses of the remaining tumor tissue from the individuals used for transcriptome analyses were consistent with a proliferative, primitive neuroectodermal-like tumor (Fig. 1C-L; Fig. S1). In the diencephalon/pretectal region of wild-type adult zebrafish brain, Olig2, Sox2 and Sox10 immunolabeling is normally restricted to cells lining the ventricles and regions adjacent to the ventricular zone (Fig. 1C-G), where stem and progenitor cells are located. Immunolabeling of zebrafish *rb1* tumors confirmed high levels of expression of Olig2, Sox2 and Sox10 and numerous phosphohistone H3-positive mitotic cells scattered throughout the lesion (Fig. 1H-L). Together, these results demonstrate the zebrafish *rb1* brain tumors have a highly proliferative, poorly differentiated state with an oligoneural precursor molecular signature found in human *OLIG2+*/*SOX10+* and zebrafish *NRAS* CNS-PNETs.

### Zebrafish *rb1* is required cell autonomously for neural precursor cell cycle exit and terminal differentiation during development

Transcriptome and molecular analysis of zebrafish *rb1* tumors indicated that an oligoneural precursor phenotype drives tumor proliferation. To identify additional molecular and genomic changes that underlie transformation and oncogenesis in the absence of RB1, we generated RNA-Seq libraries from larval zebrafish homozygous for a recessive loss of function *rb1* mutation. The *rb1/rb1* mutant transcriptome was used for comparative analysis with the tumor transcriptome to identify molecular pathways that distinguish transformed *rb1* tumor cells from nontransformed *rb1/rb1* mutant cells.

**Fig. 2.**
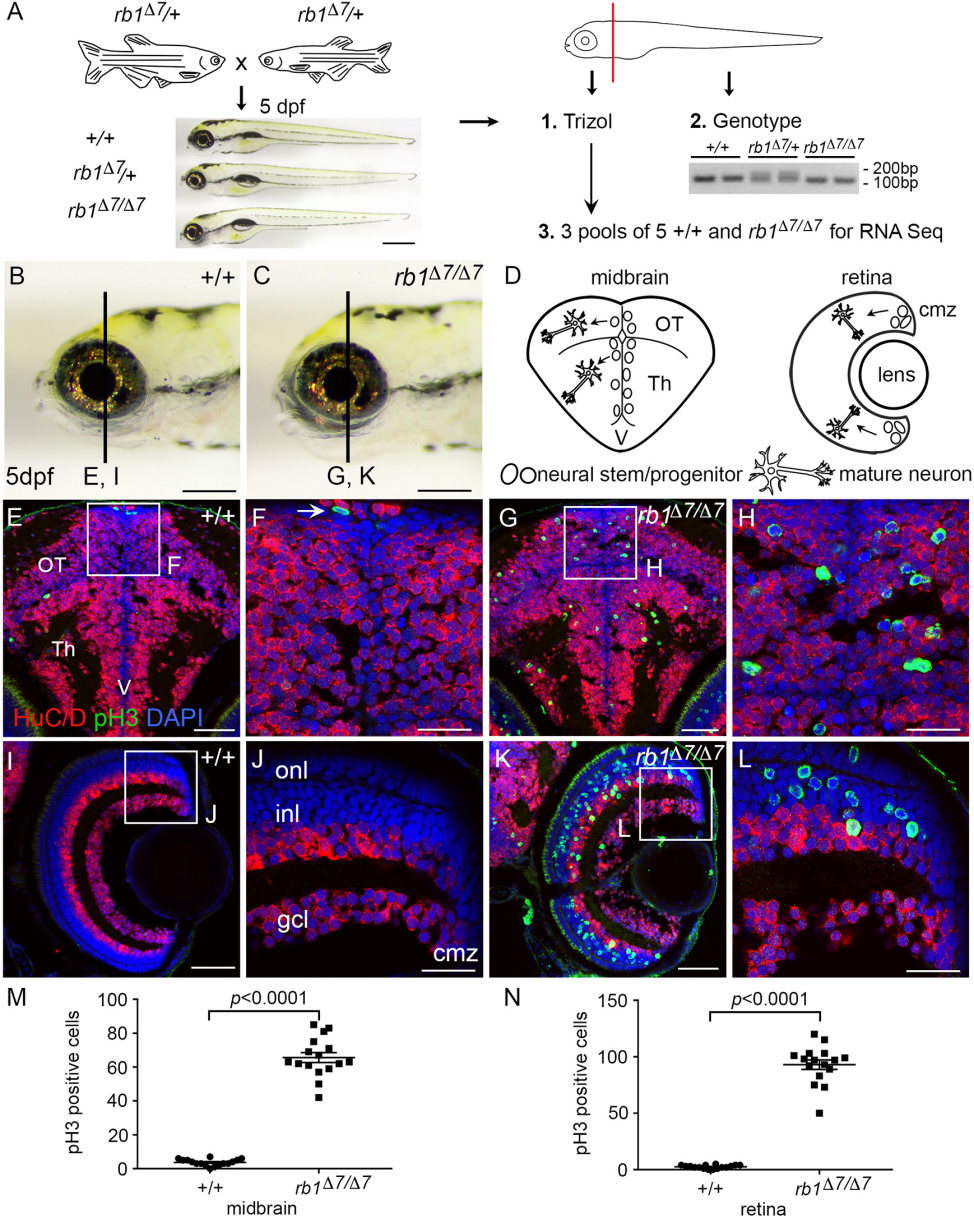
**Zebrafish *rb1Δ7/Δ7* homozygous mutant larval brain transcriptome and neurogenic phenotype.** (A) Zebrafish *rb1/rb1* mutant transcriptome was generated from *rb1Δ7/Δ7* homozygous and +/+ wild-type siblings from a cross between heterozygous *rb1Δ7/+* adults. Heads from 5 dpf larva were dissected and trunk tissue genotyped. Three pools of five heads of each genotype were used to generate RNA-Seq libraries. (B,C) Wild-type (B) and *rb1Δ7/Δ7* (C) 5 dpf larvae. (D) Diagram of larval midbrain and retina with location of neural stem and progenitor cells at brain ventricle (V) in the optic tectum (OT) and thalamic region (Th), and at the retina ciliary marginal zone (cmz). Mature, postmitotic neurons are located in the lateral brain parenchyma and inner retina. (E-L) Immunolocalization with neural differentiation marker HuC/D and mitotic M-phase marker phosphohsitone H3 (E,F,I,J). Wild-type brain and retina show the organization of mature neurons in dorsal brain optic tectum (OT), ventral brain thalamus (Th), and laminated layers of the retina (gcl, ganglion cell layer; inl, inner nuclear layer; onl, outer nuclear layer). Only one mitotic cell is detected at the brain ventricle (F, arrow). Mutant brain shows M-phase cells scattered throughout the midbrain tectum and thalamus (G,H). In the retina, numerous M-phase cells are present across the entire inner nuclear layer, with occasional cells in the outer nuclear and ganglion cell layer (K,L). (M,N) Quantification of pH3-positive cells in midbrain (M) and retina (N) sections from +/+ and *rb1Δ7/Δ7* individual 5 dpf larvae (*n*=16 for each genotype). Data are mean±s.e.m. *P*-values calculated by two-tailed unpaired Student's *t*-test. Scale bars: 200 µm (B,C); 50 µm (E,I,G,K); 20 µm (F,J,H,L).

We previously isolated a 7 bp frameshift mutation in *rb1* exon 2, *rb1Δ7^is54^*, and found that the allele is homozygous lethal during the larval stage between 5 and 10 days postfertilization (dpf) (Solin et al., 2015). To generate the *rb1Δ7/Δ7* transcriptome, 5 dpf larvae were collected from an incross between two heterozygous *rb1Δ7/+* adults. *rb1Δ7/Δ7* homozygotes do not show a dramatic gross morphological difference from wild-type or heterozygous siblings, other than the absence of a swim bladder. To confirm larval genotypes, larvae were dissected through the hindbrain; the head was placed in TRIzol and the trunk tissue used for genotyping (Fig. 2A). Five confirmed wild-type +/+ and homozygous *rb1Δ7/Δ7* heads were pooled in triplicate and used to prepare RNA-Seq libraries (Fig. 2A; Fig. S5, Table S4). Differential gene expression analysis of the *rb1/rb1* mutant larval transcriptome (Tables S5 and S6) showed upregulation of E2F targets driving cell cycle entry and IPA pathways controlling cell division, mitosis, and DNA replication, recombination and repair (Fig. S6), as expected for activation of E2F-dependent pathways in the absence of RB1. The results were remarkably similar to the *rb1* brain tumor transcriptome, suggesting that the activation of molecular pathways driving cell proliferation is controlled by similar mechanisms in *rb1/rb1* mutant and *rb1*-transformed cells.

To confirm the proliferative phenotype of *rb1/rb1* mutant cells, brain sections of 5 dpf *rb1/rb1* mutant larvae were labeled with proliferation and differentiation markers and showed an increase in mitotic-phase cells in the developing brain and retina (Fig. 2B-L). In wild-type midbrain, cycling cells are restricted to proliferative zones at the dorsal surface, ventricles and lateral edges of the brain (Fig. 2E,F), and at the ciliary marginal zone at the periphery of the retina (Fig. 2I,J), but are rarely captured in M phase by phosphohistone H3 labeling (Fig. 2E,F, boxed region and arrow, respectively). Phosphohistone H3-labeled cells are absent from the brain parenchyma (Fig. 2F) and mature retina (Fig. 2J), where the cell bodies of postmitotic differentiated neurons expressing the neuronal RNA binding protein HuC/D-Elavl3 are located in wild-type brain tissue. In contrast, in the *rb1/rb1* mutant larvae, ectopic phosphohistone H3-positive cells were detected throughout the midbrain optic tectum and thalamus (Fig. 2G,H), and in the inner and outer nuclear layers of the mature retina (Fig. 2K,L), at numbers significantly greater than in wild type **(**Fig. 2M,N; Table S7). Ectopic proliferation in the *rb1/rb1* mutant was also detected in the forebrain, cerebellum and hindbrain, along the entire anterior-posterior axis (Fig. S7). Close examination of the *rb1/rb1* mutant phosphohistone H3-positive cells showed no labeling with HuC/D (Fig. S8), suggesting that the cells were in a cycling progenitor-like state. These results are consistent with a requirement for RB1 in neural precursor cell cycle exit and terminal differentiation.

**Fig. 3.**
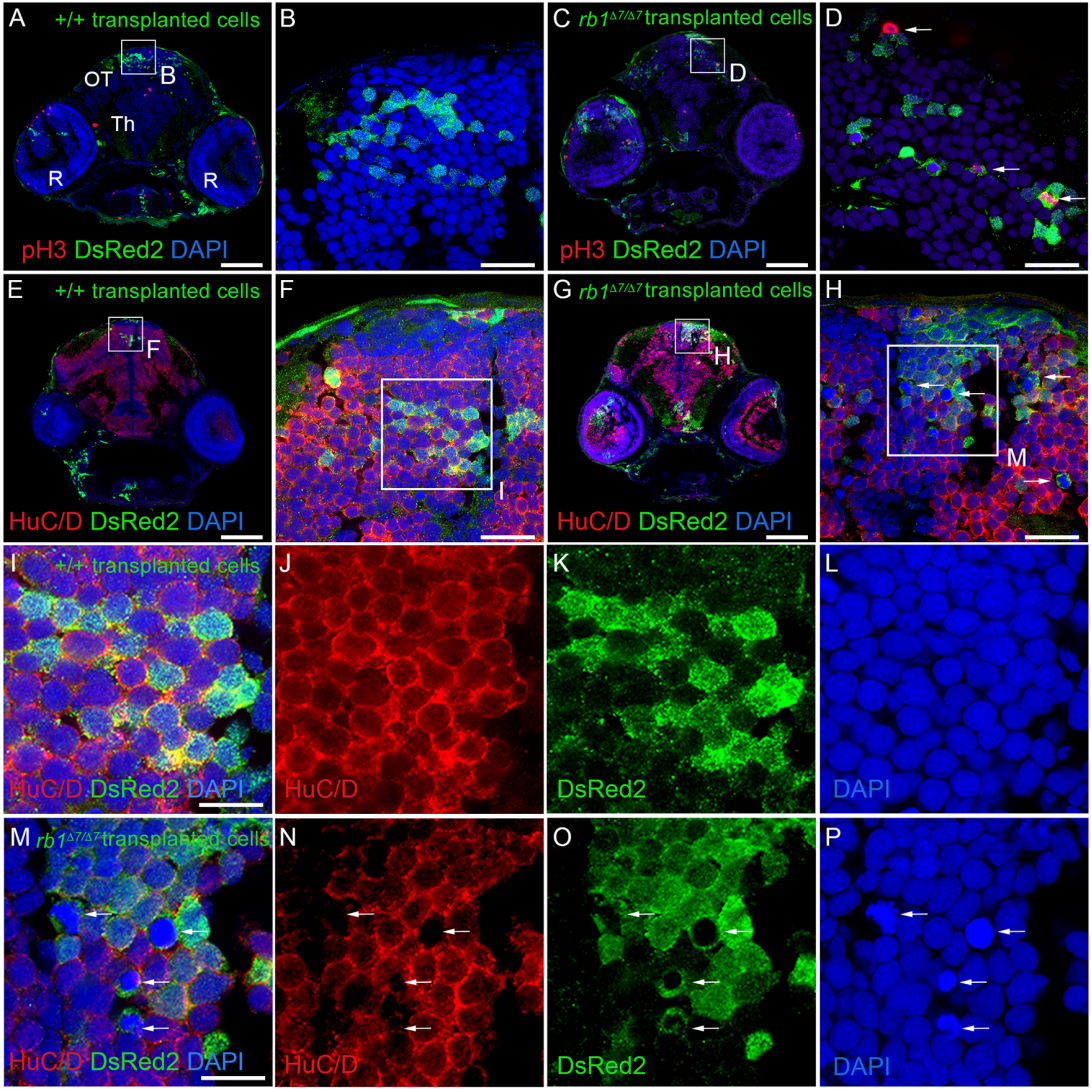
**Cell-autonomous requirement for RB1 in blocking cell cycle entry in zebrafish neural precursors.** Immunolabeling of 5 dpf host larval brain sections containing descendants of +/+; *ubi:DsRed2* and *rb1Δ7/Δ7; ubi:DsRed2* donor transplanted cells. *n*=3 individual transplant experiments for each donor genotype. (A-D) Phosphohistone H3- and DsRed2-labeled sections through the midbrain optic tectum (OT), thalamus (Th) and retinas (R). Wild-type (A,B) and *rb1Δ7/Δ7* mutant (C,D) cells present in a wild-type host optic tectum (OT). A few phosphohistone H3-positive *rb1Δ7/Δ7* mutant cells can be detected (D, arrows). (E-H) Neuronal marker HuC/D- and DsRed2-labeled sections. Wild-type (E,F) and *rb1Δ7/Δ7* mutant (G,H) cells in a host optic tectum express HuC/D. A number of *rb1Δ7/Δ7* mutant cells with highly condensed chromatin lack HuC/D labeling (H, arrows). (I-J) Boxed region in F, showing HuC/D- and DsRed2-positive wild-type cells with interphase chromatin. (M-P) Boxed region in H, showing that DsRed2-positive *rb1Δ7/Δ7* mutant cells with interphase chromatin also express HuC/D. HuC/D is absent from *rb1Δ7/Δ7* mutant cells with highly condensed chromatin (arrows). Scale bars: 100 µm (A,C,E,G); 20 µm (B,D,F,H); 10 µm (I,M).

To examine whether the requirement for RB1 in neural precursor cell cycle exit was cell autonomous, genetic mosaics were created by transplanting *rb1/rb1* mutant cells from blastula-stage embryos into wild-type host embryos and examining their behavior in larval brain. Embryos from heterozygous *rb1Δ7/+* crossed to heterozygous *rb1Δ7/+;Tg(Tol2<ubi:DsRed2>)* adults carrying a ubiquitous RFP reporter transgene were collected, and cells from blastula-stage embryos were transplanted into a *casper* host embryo. The remaining donor embryo tissue was used to determine the *rb1* genotype (Fig. S9). Three host larvae containing cells from a +/+ or *rb1Δ7/Δ7* donor embryo were analyzed. At 5 dpf, host larvae were sectioned and co-labeled with antibodies against DsRed2, to identify the descendants of transplanted cells, or the proliferation marker phosphohistone H3 or the neuronal differentiation marker HuC/D (Fig. 3). The descendants of +/+ wild-type (Fig. 3A,B,E,F) and *rb1Δ7/Δ7* mutant (Fig. 3C,D,G,H) transplanted cells were incorporated into the developing brain and could be detected in the midbrain parenchyma. None of the +/+ wild-type cells were labeled with phosphohistone H3 (Fig. 3A,B), and each cell was positive for the neuronal marker HuC/D (Fig. 3E,F). The majority of the *rb1Δ7/Δ7* mutant cells were phosphohistone H3 negative. However, in each section, one or two phosphohistone H3-positive mutant cells with highly condensed chromatin were detected (Fig. 3C,D), indicating that the cells were in M phase. Each of the *rb1Δ7/Δ7* mutant cells expressed HuC/D, except for the M-phase cells with condensed chromatin (Fig. 3G,H, arrows). Higher magnification images show that each wild-type (Fig. 3I-L) or *rb1Δ7/Δ7* mutant (Fig. 3M-P) cell that labels positively for HuC/D and DsRed2 contains chromatin characteristic of interphase cells. In contrast, the *rb1Δ7/Δ7* mutant cells with highly condensed chromatin do not co-label with HuC/D (Fig. 3M-P). The presence of *rb1Δ7/Δ7* cells that express HuC/D/Elavl3 and *rb1Δ7/Δ7* cells with condensed chromatin in M phase suggests the mutant neural precursors are able to re-enter the cell cycle. These results demonstrate that the requirement for RB1 to block cell cycle entry in neural precursors is cell autonomous.

### Comparison of zebrafish *rb1* tumor and *rb1/rb1* mutant transcriptomes suggests that epigenetics drive *rb1* tumor growth

The *rb1Δ7/Δ7* mutant transcriptome and phenotypic analyses showed that *rb1/rb1* mutant cells can re-enter the cell cycle and proceed into M phase, but lack continuous unregulated proliferation characteristic of *rb1*-transformed tumor cells. To gain insight into the molecular differences between *rb1/rb1* mutant and *rb1*-transformed cells, we performed additional comparative analyses between the zebrafish *rb1* mutant and brain tumor transcriptomes. We focused on transcriptional regulators which include epigenetic chromatin remodelers and transcription factors, because a number of studies indicate that regulation of the epigenome is a critical component of oncogenesis (Suva et al., 2013).

**Fig. 4.**
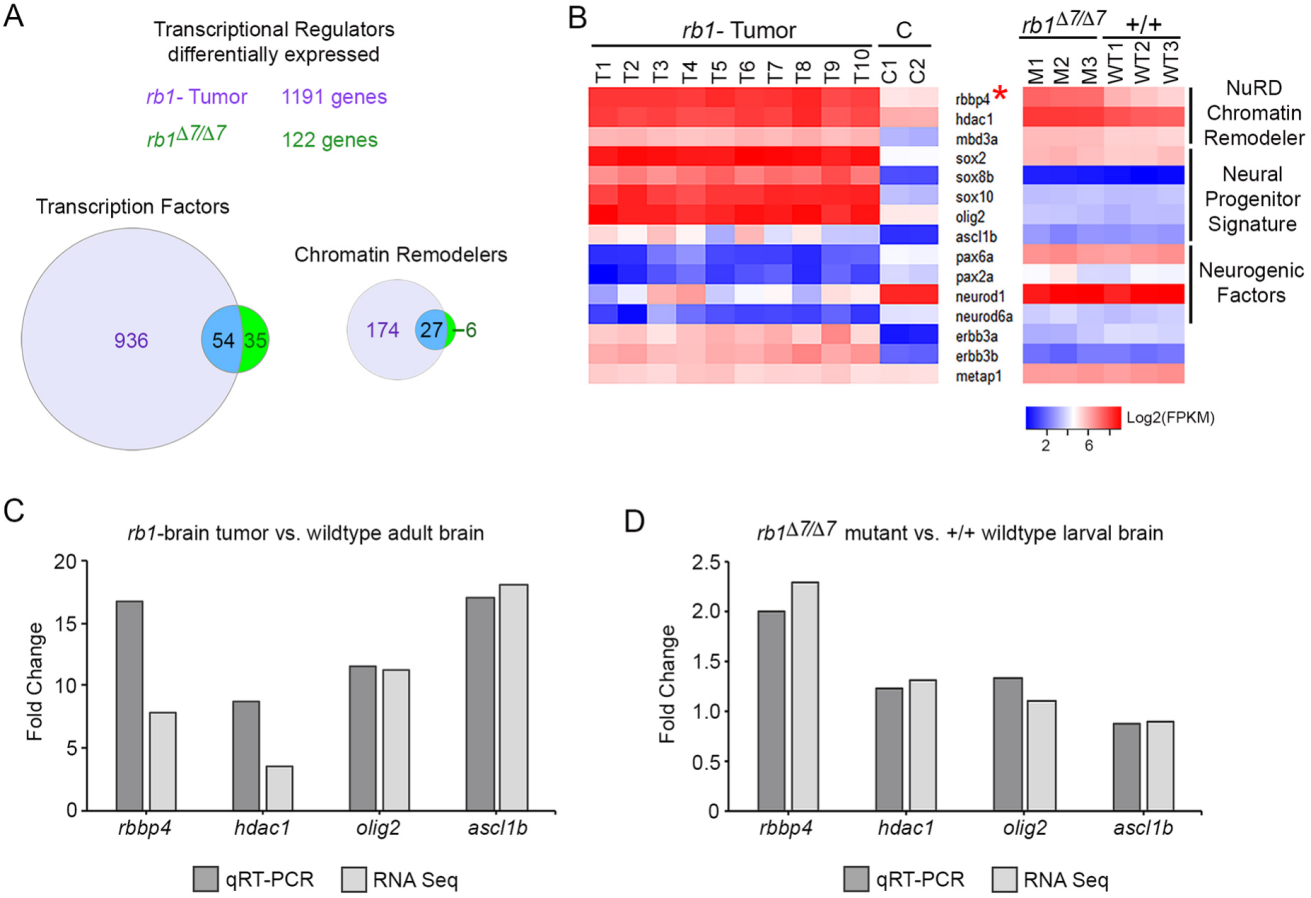
**Differential gene expression of transcriptional regulators in *rb1* tumor and *rb1/rb1* mutant transcriptomes.** (A) Comparative analysis of all differentially expressed transcriptional regulators in *rb1* tumor and *rb1/rb1* transcriptomes. (B) Heat maps of log2(FPKM) showing relative expression of components of the NuRD chromatin remodeler, neural progenitor and neurogenic transcription factors in *rb1* tumor and *rb1/rb1* mutant transcriptomes. Tables S3 and S6 contain fold change and *P*-values calculated using DESeq2. (C,D) Average fold change of *rbbp4*, *hdac1*, *olig2* and *ascl1b* measured by qRT-PCR in triplicate (dark-gray bars) and transcriptome DESeq2 calculated values (light-gray bars) in *rb1* tumor tissue versus normal adult brain (C) and 5dpf *rb1/rb1* mutant versus wild-type larvae (D). *metap1*, housekeeping gene used as a standard control for heat maps and qRT-PCR.

Of the 3302 transcriptional regulators in the zebrafish genome (Armant et al., 2013), 1191 were differentially expressed in the *rb1* tumor transcriptome, and 122 were differentially expressed in the *rb1/rb1* mutant transcriptome (Tables S3 and S6). The majority of the differentially expressed transcriptional regulators in both transcriptomes were transcription factors; approximately one fifth were chromatin regulators (Fig. 4A). However, elevated expression of stem and neural progenitor transcription factors *sox2*, *sox8*, *sox10*, *olig2* and *ascl1b*, and downregulation of proneurogenic transcription factors *pax6a*, *pax2a*, *neurod1* and *neurod6a*, was unique to the tumor (Fig. 4B). *olig2* [tumor 11.2-fold, Benjamini-Hochberg adjusted *P*-value (*P*adj)<0.00001; mutant 1.1-fold, *P*adj= 0.71904] and *ascl1b* (tumor 18-fold, *P*adj<0.00001; mutant 0.9-fold, *P*adj=0.69777) levels were confirmed by quantitative reverse transcription polymerase chain reaction (qRT-PCR) (Table S8) in *rb1* tumor tissue compared with normal adult brain (Fig. 4C), and in the 5 dpf *rb1/rb1* mutant larvae compared with wild type (Fig. 4D).

The chromatin adaptor *rbbp4*, a component of multiple chromatin remodelers controlling gene expression including NuRD, PRC2, histone acetyltransferase p300 and the cell cycle DREAM/MuvB complex, was upregulated in both *rb1* tumor (7.8-fold, *P*adj<0.00001) and *rb1/rb1* mutant (2.3-fold, *P*adj<0.00001) transcriptomes (Fig. 4B, asterisk; Tables S3 and S6). Like *rbbp4*, *hdac1*, the catalytic component of NuRD, and *mbd3a*, a DNA binding subunit, were both significantly overexpressed in the *rb1* tumor (*hdac1* 3.6-fold, *P*adj<0.00001; *mbd3a* 6.1-fold, *P*adj<0.00001). Although *hdac1* and *mbbd3a* were also upregulated in the *rb1/rb1* mutant (*hdac1* 1.3-fold, *P*adj=0.00123; *mbd3a* 1.2-fold, *P*adj=0.06262), neither was as highly elevated as *rbbp4*. qRT-PCR confirmed the change in gene expression for *rbbp4* and *hdac1* in *rb1* tumor tissue (Fig. 4C) and in the 5 dpf *rb1/rb1* mutant larvae (Fig. 4D). The differences in gene expression between tumor and mutant suggest that expression of neural stem and progenitor transcription factors, together with altered chromatin remodeler activity, correlates with transformation of *rb1* mutant cells, maintenance of the tumor progenitor-like state and tumor oncogenesis.

### Distinct requirements for chromatin remodelers *rbbp4* and *hdac1* in neurogenesis

**Fig. 5.**
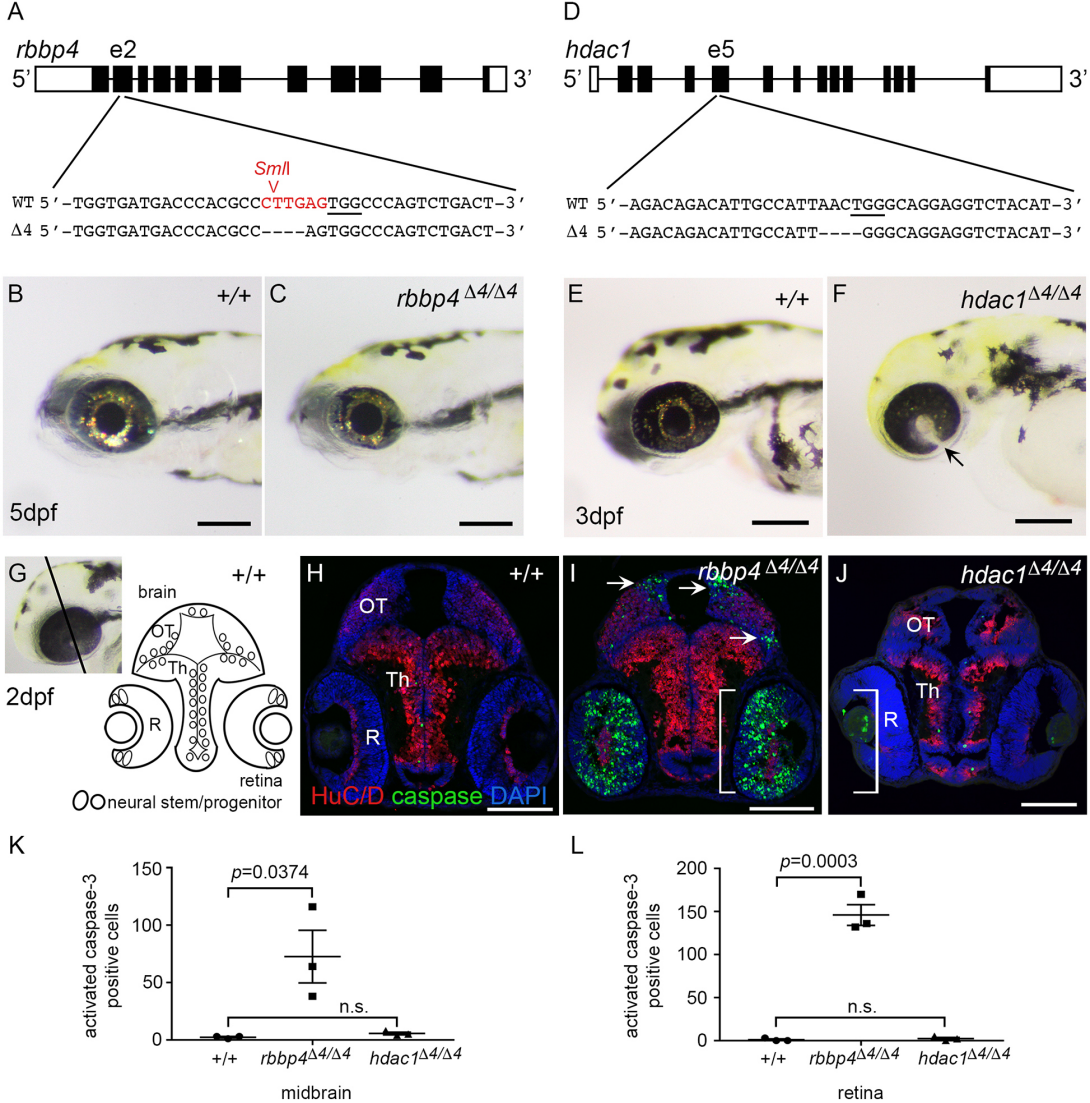
**Requirement for chromatin remodelers *rbbp4 and hdac1* in zebrafish neurogenesis.** (A) CRISPR target site in exon 2 of *rbbp4* used to isolate 4 bp frameshift mutation *rbbp4Δ4-is60*. PAM sequence is underlined. *Sml*I restriction enzyme site overlapping the target site is shown in red. (B) 5 dpf wild-type (+/+) larva. (C) Gross phenotype of 5 dpf homozygous mutant *rbbp4Δ4/Δ4* larva showing microcephaly and microphthalmia. (D) CRISPR target site in exon 5 of *hdac1* used to isolate 4 bp frameshift mutation *hdac1Δ4-is70*. PAM sequence is underlined. (E) 3 dpf wild-type larva. (F) Gross phenotype of 3 dpf homozygous mutant *hdac1Δ4/Δ4* larva showing microcephaly and retinal coloboma (arrow). (G) Diagram of 2 dpf larval midbrain and retina. (H-J) Sections of wild-type (H), *rbbp4Δ4/Δ4* (I) and *hdac1Δ4/Δ4* (J) 2 dpf larval heads labeled with neural differentiation marker HuC/D (red) and apoptosis marker activated caspase 3 (green). In *rbbp4Δ4/Δ4*, apoptosis is present in the dorsal and lateral tectum (arrows), and throughout the inner retina (brackets) (I). *hdac1Δ4/Δ4* larval brain and retina are smaller than wild type, but few apoptotic cells are detected in the brain or retina (J). (K,L) Quantification of activated caspase 3 in the midbrain (K) and retina (L) of 2 dpf wild-type, *rbbp4Δ4/Δ4* and *hdac1Δ4/Δ4* larvae. OT, optic tectum; Th, thalamus; R, retina. Scale bars: 500 µm (B,C); 200 µm (E,F); 100 µm (H-J).

To investigate how *rbbp4* and *hdac1* drive zebrafish *rb1* brain tumorigenesis, we isolated stable germline loss of function mutations in each and examined their role in neural development. CRISPR-Cas9 gene editing was used to generate a frameshift mutation in exon 2 of *rbbp4* (Fig. 5A-C) and exon 5 of *hdac1* (Fig. 5D-F). A recessive lethal loss-of-function 4 bp deletion mutation line was established for each, designated *rbbp4Δ4^is60^* and *hdac1Δ4^is70^.* Homozygous mutant *rbbp4Δ4/Δ4* larvae are lethal between 5 and 10 dpf and show a severe neurogenic phenotype with microcephaly and microphthalmia (Fig. 5C). Similar to the previously published *colgate* (*hdac1*) mutant (Henion et al., 1996; Ignatius et al., 2008), homozygous mutant *hdac1Δ4/Δ4* are also larval lethal, and at 3 dpf show a reduced body size, curved trunk, microcephaly and coloboma in the retina (Fig. 5F, arrow). Homozygous mutant larvae were examined at 2 dpf to determine the defect underlying the reduced size of the brain and retina (Fig. 5G-J). In 2 dpf wild-type larval brain and retina, only a few apoptotic cells were labeled with an antibody against activated caspase 3 (Fig. 5H). *rbbp4Δ4/Δ4* mutants showed extensive apoptosis in the midbrain optic tectum and retina, regions of active proliferation as the embryo transitions to larval neurogenesis, demonstrating that Rbbp4 is required for survival of neural precursors (Fig. 5I). The failure of *rbbp4Δ4/Δ4* neural precursors to survive explains the reduced eye and microcephaly observed in mutant larvae. In contrast to *rbbp4*, which showed significant high levels of apoptotic cells, the amount of apoptosis in the 2 dpf *hdac1Δ4/Δ4* mutant was not statistically different from wild type (Fig. 5J,K,L; Table S9). However, the size of the tectum and thalamus was reduced compared with wild type, with a decrease in the amount of HuC/D-expressing neurons in the parenchyma (Fig. 5J). The absence of apoptosis combined with an overall reduced size suggests that *hdac1* is required for persistent proliferation of stem cells to generate the neural progenitor pool and new neurons. These results show a distinct requirement for *rbbp4* in neural progenitor or precursor survival, whereas *hdac1* is necessary for maintaining proliferation of the neural stem and/or progenitor pool. Similar roles in neural progenitor proliferation and survival could drive unregulated tumor growth in zebrafish *rb1* brain tumors.

### Somatic targeting assay to examine the requirement for chromatin remodelers in neural stem and progenitor cells during development

**Fig. 6.**
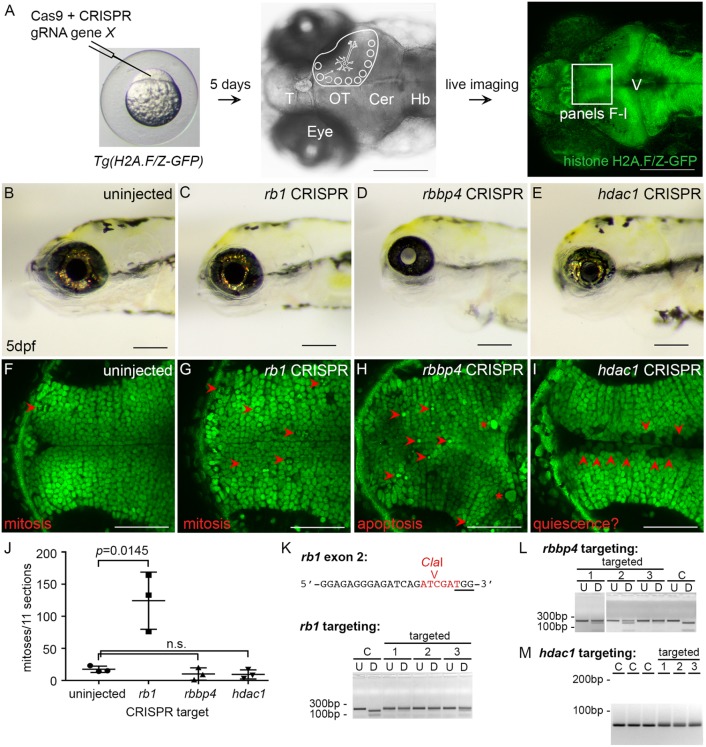
**CRISPR somatic targeting-live imaging assay to test chromatin remodeler function in neural cell populations during brain development.** Somatic inactivation recapitulates germline mutants and reveals distinct requirements for *rb1*, *rbbp4* and *hdac1* in neural progenitor proliferation, differentiation and survival. (A) CRISPR targeting in histone H2A.F/Z-GFP fusion line and dorsal view, and confocal live imaging of GFP in the brain at 5 dpf. (B) 5 dpf wild-type larvae. (C-E) Gross defects in 5 dpf *rb1* (C), *rbbp4* (D) and *hdac1* (E); CRISPR targeted larvae recapitulate germline phenotypes. (F) Confocal imaging in the optic tectum in the brain of a live 5 dpf larva. Section through the ventral region of the optic tectum. Mitotic figures are rarely captured in uninjected larvae at this level of the tectum (red arrowhead). (G) Tectum in *rb1* CRISPR-targeted larva shows cells with nuclei containing condensed chromatin (red arrowheads) scattered throughout the postmitotic region of the tissue. (H) Tectum in *rbbp4* CRISPR-targeted larva shows intense, punctate fluorescence in presumed apoptotic nuclei (red arrowheads) and abnormally large, irregular nuclei (asterisks). (I) Tectum in *hdac1* CRISPR targeted larva shows an overall reduction in size and cell number. Enlarged nuclei with faint GFP line in the ventricle (red arrowheads) are observed. (J) Quantification of M-phase phosphohistoneH3-positive nuclei in the optic tectum in three uninjected control, *rb1*-, *rbbp4*- and *hdac1*-targeted larvae. Targeting experiments were replicated three times for *rb1*, and two times for *rbbp4* and *hdac1*. Plot shows mean±s.e.m. number of positive nuclei in 11 sections, in three biological replicates. The number of M-phase nuclei in *rb1*-targeted larvae is significantly different from that in control larvae. *rb1*, *P*=0.0145; *rbbp4*, *P*=0.3024; *hdac1*, *P*=0.1841; n.s., nonsignificant (two-tailed unpaired Student's *t*-test). (K-M) PCR genotyping shows highly efficient mutagenesis in the three CRISPR-targeted individuals used for quantification. C, control uninjected individuals. Location of *rb1* exon 2 CRISPR gRNA and *Cla*I enzyme site overlapping Cas9 cut site (K). U, undigested PCR amplicon; D, *Cla*I-digested PCR amplicon surrounding exon 2. *rbbp4* and *hdac1* targeting was performed with the gRNAs used to isolate loss of function alleles described in Fig. 5. *rbbp4* targeting (L). U, undigested exon 2 PCR amplicon; D, *Sml*I-digested exon 2 PCR amplicon. In the *hdac1*-targeted larvae the diffuse PCR amplicon band on the gel in targeted individuals correlates with efficient mutagenesis (M). Scale bars: 200 µm (A-E); 50 µm (F-I).

CRISPR-Cas9 somatic targeting in zebrafish provides a simple and rapid method to investigate the function of a gene during embryonic and larval development. We developed an *in vivo* live animal imaging assay to examine the role of chromatin remodelers and other candidate tumor suppressor genes in neural stem and progenitor cells during brain development (Fig. 6A). CRISPR-Cas9 somatic targeting is performed in the *Tg(H2A.F/Z-GFP)* reporter line (Pauls et al., 2001), which expresses a histone H2A.F/Z-GFP fusion protein that labels chromatin in all nuclei. We validated the assay by somatic targeting of *rb1*, *rbbp4* and *hdac1*, and comparing the phenotype in live 5 dpf larvae with the fixed immunohistochemical analysis of homozygous germline mutants (Fig. 6C-E). CRISPR targeting of *rbbp4* and *hdac1* resulted in gross defects in the head, consistent with the phenotype of stable germline loss of function mutants described above (Fig. 6D,E).

To examine the effects of somatic targeting on neurogenesis at the cellular level, we imaged the optic tectum in live *Tg(H2A.F/Z-GFP)* 5 dpf uninjected **(**Fig. 6F), *rb1* CRISPR-targeted (Fig. 6G), *rbbp4* CRISPR-targeted (Fig. 6H) and *hdac1* CRISPR-targeted (Fig. 6I) larvae. Larvae were mounted with dorsal side upright. A confocal z-stack of 11 slices was collected at 3 μm steps, beginning ∼30 μm below the surface of the tectum, moving ventrally towards the thalamic region. In this ventral region of the tectum, the rate of stem cell division has slowed dramatically compared with the actively proliferative zone at the dorsal side. In wild-type larvae, a single confocal slice through the ventral tectum shows the highly organized radial pattern of neuronal nuclei extending laterally away from the midline. A clear demarcation of cells lining the ventricle marks the midline (Fig. 6F). In the larval midbrain, stem cells line the ventricles and the anterior-lateral surface of the tectum. In the confocal section shown, a single metaphase mitotic figure is present near the anterior side of the uninjected wild-type tectum (Fig. 6F, arrowhead). Quantification of mitotic figures in 11 wild-type sections showed 14-23 dividing cells per individual tectum (*n*=3) (Fig. 6J; Table S10).

Live imaging of the tectum in *rb1* CRISPR-targeted larvae showed a dramatic increase in the number of cells with condensed chromatin (Fig. 6G, arrowheads), consistent with the phosphohistone H3 immunolabeling results from *rb1/rb1* mutant fixed, sectioned tissue. The M-phase cells were located near the ventricle and also distributed throughout the tectum, where the regular array of postmitotic neuron cell bodies are located. The total number of cells with condensed chromatin in *rb1*-targeted embryos was significantly higher than in wild type, ranging from 76 to 133 (*n*=3, *P*=0.0145) (Fig. 6JD; Table S8). The variability in total number in *rb1*-targeted larvae might be caused by the degree of mosaicism and formation of bi-allelic indel mutations, although genotyping indicated that each was efficiently mutagenized at the CRISPR target site (Fig. 6K). These results suggest that *rb1/rb1* mutant neural precursors can re-enter the cell cycle after migration into the postmitotic region of the tectum.

To examine the behavior of *rb1* mutant neural precursors in real time, time-lapse confocal images were captured of H2A-GFP in wild-type and homozygous mutant *rb1Δ7/Δ7* larval brains (Movies 1 and 2, Fig. S9B). Live imaging of the dorsal surface of the optic tectum in wild type at 3 dpf (Movie 1) and 5 dpf (Movie 2) shows that the tissue is highly proliferative, with multiple stem cells entering and completing mitosis. At 5 dpf, in more ventral regions of the tectum where tissue growth has slowed, a 2-h time lapse showed quiescent neural precursors, and only one stem cell at the periphery was captured undergoing mitosis (Movie 3). In contrast, in the *rb1Δ7/Δ7* mutant, multiple cells throughout the tectum underwent chromosome condensation (Movie 4). Over the course of 3 h, cells that had entered the cell cycle failed to progress through metaphase, and frequently appeared to be removed by microglia. These analyses confirmed in real time the requirement for zebrafish Rb1 in blocking neural precursor cell cycle re-entry, and support an additional role for *rb1* in promoting metaphase progression and completion of mitosis.

The role of zebrafish Rbbp4 and Hdac1 in neural cell populations during neurogenesis was also examined by live imaging in CRISPR-targeted larvae. Quantification confirmed that proliferation in the tectum was not significantly different from wild type after targeting *rbbp4* (1-9 cells per larva; *n*=3, *P*=0.3024) (Fig. 6H,J,L; Table S8). In *rbbp4*-targeted larvae, cell nuclei throughout the tectum appeared fragmented or hypercondensed, reminiscent of cells undergoing apoptosis (Fig. 6H, arrowheads). Frequently, very enlarged nuclei or cells were detected near the tectum posterior border (Fig. 6H, asterisks). Like the analysis of fixed tissue from homozygous mutants, live imaging of the *rbbp4*-targeted larval brain revealed that *rbbp4* is required for survival of newborn or postmitotic neurons.

*hdac1* CRISPR-targeted larvae (Fig. 6I,M) also showed a consistent neurogenic phenotype, but a range of severities. In some larvae the optic tectum was reduced in size, while in others the midbrain ventricle was completely open and tectal structures entirely missing. Imaging of *hadc1*-targeted larvae that had developed an optic tectum revealed no significant difference in the number of mitotic cells compared with wild type (3-17 cells per larva; *n*=3, *P*=0.1841) (Fig. 6I,J; Table S8). However, the nuclei in cells lining the ventricle appeared enlarged, with diffuse GFP expression (Fig. 6I, arrowheads), indicating that the chromatin was decondensed. The reduced size of the brain, together with decondensed chromatin in cells lining the ventricle, suggests that the neural stem cells in the *hdac1*-targeted larvae had exited the cell cycle and become quiescent. These results support a requirement for *hdac1* in maintenance of a proliferating neural stem cell population.

**Fig. 7.**
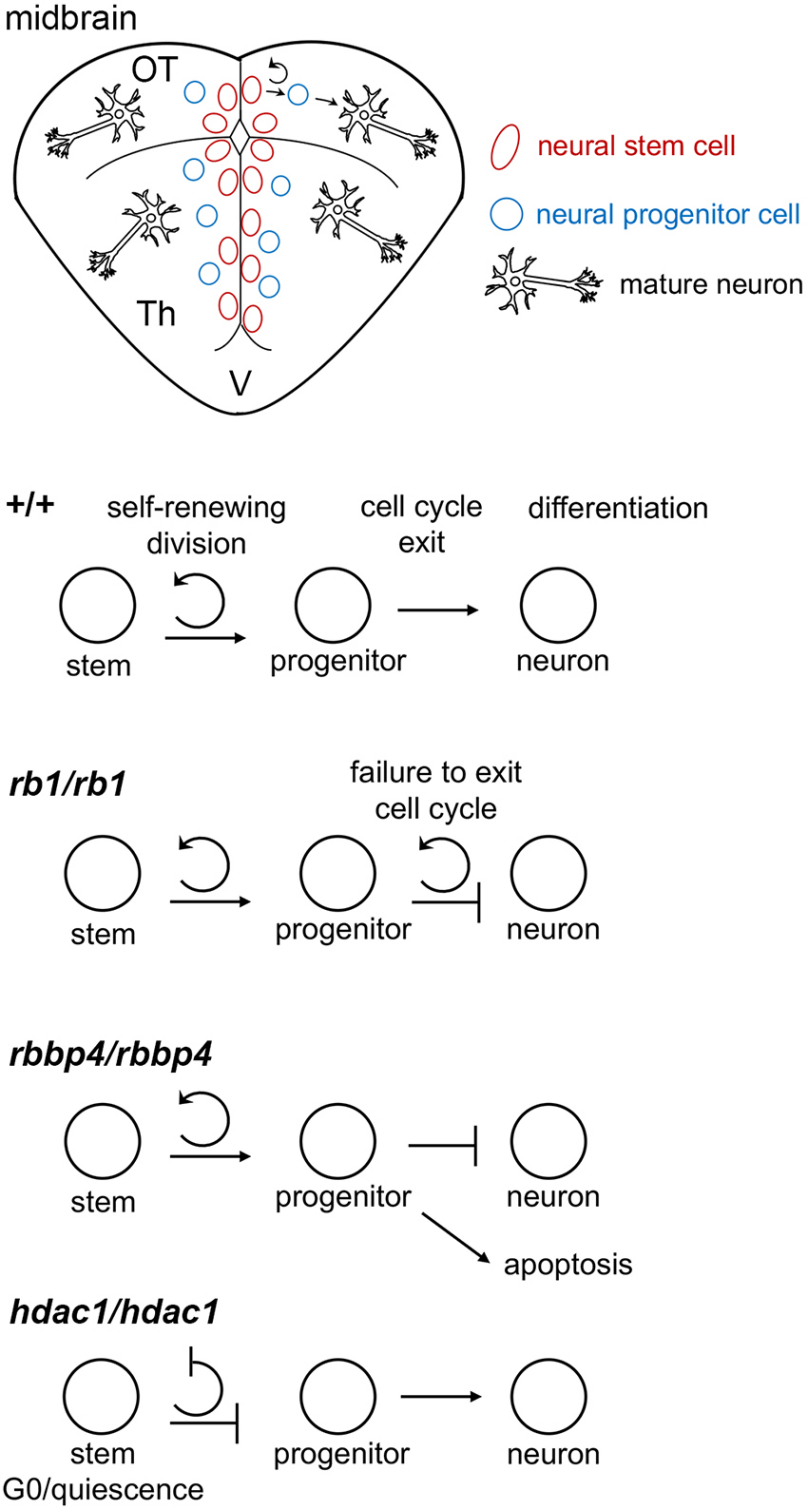
**Roles for *rb1* and chromatin regulators *rbbp4* and *hdac1* in proliferation, survival and terminal differentiation during neurogenesis.** Diagram of spatial organization of neurogenesis in the zebrafish midbrain. In the midbrain, stem cells in the optic tectum (OT) and thalamus (Th) line the ventricles and undergo self-renewing divisions to produce progenitors. Progenitors exit the cell cycle, and neural precursors migrate to their final destination in the parenchyma, where they differentiate into mature neurons. Germline mutant and CRISPR somatic-targeting analyses showed that zebrafish *rb1* is required cell autonomously to prevent neural precursor cell cycle re-entry. *rbbp4* is required for survival of neural precursors. *hdac1* is necessary to maintain proliferation in the stem cell pool, possibly by preventing premature cell cycle exit and differentiation, and/or quiescence.

Our CRISPR somatic targeting-live imaging assay is a simple, rapid method to test the function of transcriptional regulators in neural cells and gain additional insight into their roles in tumor cell proliferation and oncogenesis. The somatic targeting assay confirms the germline mutant phenotype and reveals a dynamic view of proliferation and commitment in neural cell populations in the developing midbrain (Fig. 7). Our analyses suggest that *rb1* is necessary to block re-entry of neural precursors into the cell cycle. In the absence of *rbbp4*, neural precursors fail to survive and activate caspase 3-mediated apoptosis. The production of new neural precursors is dependent on *hdac1* maintaining proliferation of the neural stem cell pool. Together, these results suggest that elevated expression of *rbbp4* and *hdac1* after *rb1* loss drive unregulated proliferation and growth in zebrafish *rb1* brain tumors.

## DISCUSSION

In this study, we compared the transcriptome of somatically engineered zebrafish *rb1* primitive neuroectodermal-like brain tumors with homozygous *rb1/rb1* mutants and identified epigenetic regulators that could contribute to the mechanism of RB1 mutant tumor oncogenesis. Our results suggest that transcriptional regulation by epigenetic modifiers and neural progenitor transcription factors distinguishes transformed RB1 mutant tumor cells from homozygous mutant brain tissue harboring a simple loss-of-function mutation in *rb1*. We next performed phenotypic mutant analysis in the developing zebrafish larval brain to understand the role of *rb1* and the chromatin remodelers *rbbp4* and *hdac1* in normal neurogenesis. As part of this analysis, we developed a novel *in vivo* assay to examine RB1 and chromatin remodelers in neural precursor proliferation, survival and differentiation. The results show a cell-autonomous requirement for *rb1* in blocking cell cycle re-entry in neural precursors. While *rbbp4* is required for neural progenitor and/or precursor survival, *hdac1* is necessary for maintaining proliferation of the neural stem/progenitor pool. The requirements for *rbbp4* and *hdac1* in regulating neural progenitor proliferation and survival might contribute to oncogenesis after *rb1* loss in zebrafish *rb1* brain tumors. Our study provides novel insights into the tumor suppressive role of zebrafish Rb1 and its candidate interacting proteins Rbbp4 and Hdac1 in driving persistent tumor growth, and identifies Rbbp4 as a potential target to inhibit tumor cell survival.

Our zebrafish *rb1* brain tumor model histologically and molecularly resembles human CNS-PNET, an embryonal tumor composed of proliferative neuroblast cells. Conditional *Rb1* knockout in mice neural precursors in combination with *Tp53 (Trp53)* and *Pten* tumor suppressors favors formation of CNS-PNET over glioma (Chow et al., 2011; Jacques et al., 2010). Although our zebrafish model results from inactivation of a single tumor suppressor *rb1*, tumor pathway analysis identified *TP53*-dependent signaling, DNA mismatch, base excision and homologous recombination repair pathways as altered in *rb1* tumors, and these factors might contribute to transformation and tumorigenesis. In humans, CNS-PNET has been genetically linked to mutation or alteration of *RB1/E2F*, *TP5*3 and DNA mismatch repair pathways (reviewed in Larson and Largaespada, 2012). It has been reported that 4-15% of children carrying *RB1* mutations develop PNETs outside of the retina (Blach et al., 1994; de Jong et al., 2015; Kivelä, 1999), and that silencing of *CDKN2A*, a negative regulator of RB1-inhibiting cyclin-dependent kinases, occurs in sporadic PNETs (Li et al., 2005; Pfister et al., 2007). Sporadic CNS-PNET has also been associated with *TP53* mutation and overexpression (Eberhart et al., 2005) and *TP53* germline mutation in Li Fraumeni syndrome (Orellana et al., 1998). A number of tumor types, including CNS-PNET, arise in individuals with Turcot syndrome Type I, which is linked to mutations in the DNA mismatch repair genes *MLH1*, *MSH2*, *PMS1* and *PMS*2 (De Vos et al., 2004). Altered expression of *RB1/E2F*, *TP53* and DNA mismatch repair pathways in the zebrafish *rb1* brain tumor model is consistent with the genetic origin of human CNS-PNETs.

Comparative genomics revealed that the zebrafish RB1 brain tumors are most similar to the human *OLIG2+/SOX10+* CNS-PNET subtype (Picard et al., 2012; Sturm et al., 2016). We confirmed that Olig2 and Sox10 are highly expressed in tumor tissue, supporting a mechanism in which oligoneural precursor transcription factors contribute to gene expression driving tumor cell proliferation. The previous observations that epigenetic mechanisms drive cell transformation and tumorigenesis in human and mouse retinoblastoma (Aldiri et al., 2017; Benavente et al., 2013; Zhang et al., 2012) suggested that epigenome changes also underlie oncogenesis in the zebrafish *rb1* tumor model. Differential gene expression showed that >36% of all transcriptional regulators, including >170 chromatin remodelers, are altered in the *rb1* tumor transcriptome, which suggests epigenetic control of gene expression drives tumorigenesis. RBBP4, a chromatin adaptor for multiple chromatin remodeling complexes, is an E2F target and, as expected, was increased in the zebrafish *rb1* tumor and *rb1/rb1* homozygous mutant transcriptome. *hdac1*, which is not a direct target of E2F transcriptional activation, was highly upregulated in the tumor transcriptome, but only elevated 1.3-fold in the *rb1/rb1* mutant. This suggests that *hdac1* is one of many chromatin remodelers for which activity might distinguish transformed tumor cells from mutant cells. Gene regulatory network analysis and simulation of tumorigenesis in a human cell line model has suggested that chromatin remodelers cooperate with transcription factors as cells progress to transformation (Malysheva et al., 2016). A similar epigenetic mechanism in which Hdac1 and Rbbp4 associate with oligoneural precursor transcription factors might drive zebrafish *rb1* brain tumor oncogenesis.

Our analysis of zebrafish loss-of-function *rb1/rb1* mutants in neural development reveals a cell-autonomous requirement for *rb*1 in suppressing cell cycle re-entry in neural precursors. This is consistent with the original mouse *Rb1* knockout models demonstrating ectopic proliferation in the embryonic mouse brain (Clarke et al., 1992; Ferguson and Slack, 2001; Jacks et al., 1992; Lee et al., 1992; Lipinski and Jacks, 1999). More recently, conditional *Rb1* knockout in embryonic and adult mouse neural progenitors has shown that *Rb1* is required to control neural precursor proliferation and for the long-term survival of newborn neurons in the olfactory bulb and hippocampus (Naser et al., 2016; Vandenbosch et al., 2016). Although our results do not indicate that zebrafish Rb1 functions in long-term neuron survival, analysis of *rb1/rb1* mutant cells over an extended time would be required to rule out this role. Our live imaging of histone H2A-GFP in zebrafish larval brain allowed a dynamic view of *rb1/rb1* mutant neural precursor behavior and cell cycle entry. Following chromatin condensation, *rb1/rb1* mutant cells failed to progress through mitosis and appeared to idle in prometaphase, indicating that RB1 is also required to complete the cell cycle. A delay in cell cycle exit of retinal ganglion cell precursors was previously reported in the zebrafish *rb1* mutant *space cadet* (Gyda et al., 2012). Whether *rb1/rb1* mutant neural precursors remain arrested in M phase, eventually exit the cell cycle, or undergo continuous rounds of inappropriate cell cycle entry will require additional analyses.

Recently, RB1 has been shown to cooperate with the DREAM complex subunit Lin37 to block cell cycle entry and transition cells from G1 to G0/quiescence (Mages et al., 2017). In budding yeast, the transition from proliferation to quiescence is mediated by DREAM recruitment of the histone deacetylase HDAC1 to S-phase genes to repress cell cycle entry (Miles and Breeden, 2017), again connecting RB1 with chromatin remodeling in cell cycle regulation. RB1 has also been shown to interact genetically with the chromatin remodeler histone demethylase UTX in cell fate control in *Caenorhabditis* *elegans* (Wang et al., 2010a). Zebrafish *rb1/rb1* mutant neural precursors fail to initiate quiescence, but our data showed this also lack the ability to respond to differentiation signals in the environment. RB1 in association with HDAC1 and other chromatin remodelers might be required to shift the gene expression program in G1 neural precursors to a neural differentiation program. The disruption of RB1-dependent transcriptional mechanisms controlling neural precursor quiescence and differentiation could be a part of oncogenesis in RB1 mutant brain tumors.

The zebrafish *rbbp4* and *hdac1* mutants we isolated and characterized have provided new insight into their roles in neural cell fate decisions. RBBP4 has not previously been studied in the developing vertebrate nervous system, and our analysis shows that *rbbp4* is necessary for neural precursor survival in zebrafish. RBBP4 acts as a chromatin adaptor for multiple chromatin regulatory complexes, including HDAC1/NuRD (Alqarni et al., 2014), PRC2 (Kuzmichev et al., 2002; Müller et al., 2002), DREAM/MuvB (Harrison et al., 2006; Litovchick et al., 2007) and the transcriptional activator p300/histone acetyl transferase (Kitange et al., 2016). In zebrafish neural precursors, Rbbp4 could assist in genome maintenance through p300 activation of gene expression in DNA repair pathways, as was reported in glioblastoma cells (Kitange et al., 2016). Additional phenotype and genetic interaction studies of zebrafish *rbbp4* are necessary to dissect the contribution of RBBP4 and its chromatin remodeling complexes to preventing neural precursor apoptosis and facilitating neural differentiation. Like RBBP4, HDAC1 is a component of multiple chromatin remodeling complexes that function separately to control proliferation and differentiation, depending on the cell type and context. The zebrafish *hdac1* mutant phenotype would support a role for HDAC1 in maintaining stem cell proliferation and the production of neural progenitors, either by blocking quiescence or premature differentiation in the stem cell population. The decondensed, enlarged nuclear phenotype of zebrafish *hdac1* mutant stem cells in the brain suggests that histone deacetylation is important for maintaining a proliferative stem cell pool. Global loss of histone acetylation owing to overexpression of HDAC proteins is a common feature in several human cancers (Haery et al. 2015). Together with the survival-promoting activity of RBBP4, HDAC1 and RBBP4 might cooperate to drive persistent proliferation and growth of tumor cells in RB1 mutant brain cancer.

Future studies to investigate the cooperation of RBBP4, HDAC1 and RB1 in specific neural precursor cell types will increase understanding of the mechanisms by which RBBP4 prevents programmed cell death and HDAC1 maintains a balance between proliferation and differentiation and/or quiescence. Since the first description of somatic targeting in zebrafish using custom genome editing nucleases (Bedell et al., 2012), CRISPR/Cas9 and TALENs have been widely adopted to examine developmental mechanisms and model disease (Peng et al., 2014; Shah et al., 2016). Combined with the histone-GFP reporter live imaging assay we developed, our somatic screen provides a rapid method to test chromatin remodelers and further dissect the mechanism controlling RB1-dependent proliferation and survival in neurogenesis and tumor suppression. The recent discovery of a WD40-repeat inhibitor that binds the MLL subunit of PRC2 (Song et al., 2017) suggests that other WDR proteins, such as RBBP4, are strong candidates for chemical compound screening to identify therapeutic drugs. Given the requirement for RBBP4 in neural precursor survival, RBBP4 and its associated chromatin remodeling complexes represent a potential new target to inhibit tumor cells and induce neural cancer cell apoptosis.

## MATERIALS AND METHODS

### Zebrafish care and husbandry

Zebrafish were raised in an Aquatic Habitat housing system from Pentair Aquatic Eco-systems and maintained at 27°C on a 14 h light/10 h dark cycle. The zebrafish WIK wild-type strain and transparent *casper* mutant line (White et al., 2008) were purchased from the Zebrafish International Resource Center (https://zebrafish.org/home/guide.php). Transgenic line *Tg(H2A.F/Z-GFP*) (Pauls et al., 2001) was received from Dr Brian Link, Medical College of Wisconsin. Isolation of the zebrafish *rb1* 7 bp exon 2 frameshift mutation *rb1Δ7is54* was described previously (Solin et al., 2015). All experimental protocols were approved by the Iowa State University (ISU) Animal Care and Use Committee (Log#11-06-6252) and performed in compliance with American Veterinary Medical Association and National Institutes of Health (NIH) guidelines for the humane use of laboratory animals in research. The zebrafish *rb1* brain tumor model was generated by injection of *rb1* TALENs targeting exon 2 (Solin et al., 2015). Animals were monitored according to the guidelines for endpoint in neoplasia studies as outlined in the NIH/Office of Animal Care and Use/Animal Research Advisory committee (ARAC) Guidelines for endpoint in neoplasia studies (oacu.od.nih.gov/ARAC/Guidelines for Endpoints in Animal Study Proposals). Adult fish were monitored daily during feeding for general appearance, size, length, viability and morbidity relative to control siblings. Fish were monitored bi-weekly for evidence of cranial tumors, and sacrificed before tumor burden reached 3 mm in size/25 mg in weight, constituting less than 10% of the total body weight of an adult fish (300-500 mg).

### RNA-Seq libraries, sequencing and analysis

#### Zebrafish *rb1* brain tumor model RNA-Seq libraries

Tissues for isolation of total RNA were dissected from ten adult fish with brain tumors and six control wild-type siblings aged 6.5-10.5 months. One sagittal half of each brain from the diencephalon to behind the cerebellum was isolated for RNA extraction. The remaining half was processed and embedded in either paraffin blocks or OCT media (Thermo Fisher Scientific). Total RNA was isolated using TRIzol (Invitrogen), followed by DNase I (Qiagen) digestion and Qiagen RNeasy MinElute Cleanup (Qiagen). RNA quality was analyzed with the Agilent RNA 6000 Nano Kit on an Agilent 2100 Bioanalyzer. One microgram of total RNA input was used for each library preparation. Replicate cDNA libraries were synthesized from ten individual brain tumor RNA samples and two pools of three wild-type brain RNA samples using the Illumina TruSeq RNA Library Prep Kit v2., for a total of 24 barcode-indexed libraries. The 24 cDNA libraries were multiplex 50 bp single-end sequenced in two lanes (12 per lane) on an Illumina HiSeq 2500 instrument at the Genome Sequencing Core, Center for Molecular Analysis of Disease Pathways, University of Kansas.

#### Zebrafish *rb1Δ7/Δ7* mutant 5 dpf larval brain RNA-Seq libraries

Heterozygous *rb1Δ7/+* adults were in-crossed and embryos aged to 5 dpf for tissue isolation. Individual 5 dpf larvae were dissected in two; the head was placed in TRIzol for storage at −80°C; the trunk was placed in 50 mM NaOH, heated at 95°C for 30 min, and used for PCR genotyping. Three pools of five genotyped heads were used to prepare three wild-type +/+ and three *rb1Δ7/Δ7* homozygous mutant barcoded indexed RNA-Seq libraries, as described above. The six indexed libraries were multiplex 50 bp single-end sequenced in two lanes on an Illumina HiSeq 2500 instrument at the Genome Sequencing Core, Center for Molecular Analysis of Disease Pathways, University of Kansas.

Reads were aligned to the GRCz10 zebrafish reference genome using GSNAP version 20150723 with the following parameters ‘-N 1 -t 8 -B 4 -m 5 -A sam --split-output’ to account for exon-exon splice junctions during alignment and a maximum of five mismatches. The ‘uniq’ SAM output files from GSNAP were funneled through Cufflinks 2.2.1 for normalization. Python HT-Seq 0.9.1 was used to generate the count table from the GSNAP ‘uniq’ SAM files. Gene exonic length was calculated from the Danio_rerio.GRCz10.89.gtf file in R using the ‘GenomicFeatures’ package from Bioconductor. Calculation of fragments per kilobase of transcript per million mapped reads (FPKM) was performed using the following equation: FPKM=(10^9^×number of mapped reads to a gene)/(gene exonic length×total mapped reads in the experiment). Count data generated from HT-Seq were analyzed for differential gene expression detection using DESeq2. Differentially expressed genes were defined by an adjusted *P*-value of less than or equal to 0.01 and a 1.5-fold change in either direction.

Correlation coefficient plots were computed using FPKM in Microsoft Excel with the CORREL function. GSEA and E2F target gene analysis was performed at the Broad Institute website (http://software.broadinstitute.org/gsea/index.jsp). IPA (830036, Qiagen) was performed with software licensed to ISU. For IPA, human homologs of zebrafish genes were identified at Biomart (http://www.biomart.org). Differential gene expression of 3302 transcriptional regulators was analyzed using the gene list identified in Armant et al. (2013). Heatmaps were generated in R with gplots heatmaps.2 or with Broad Institute GENE-E software (https://software.broadinstitute.org/GENE-E/). Venn diagrams were drawn at http://www.venndiagrams.net.

### qRT-PCR

Larvae at 5 dpf from an in-cross between *rb1Δ7/+* adults were collected and dissected into head and trunk tissue. Head tissue was placed in TRIzol. Trunk tissue was digested in 50 mM NaOH at 95°C for 30 min, then PCR genotyped to identify homozygous *rb1Δ7/Δ7* and wild-type +/+ siblings. Thirty-three homozygous *rb1Δ7/Δ7* heads and 40 +/+ heads were pooled, and total RNA extracted. mRNA was purified from total RNA using a magnetic isolation kit (E7490S, NEB) and checked for quality using an Agilent 2100 Bioanalyzer. First-strand cDNA was reverse transcribed with SuperScript III First-Strand Synthesis SuperMix for qRT-PCR (11752-050, Invitrogen). qRT-PCR primers are listed in Table S8. Quantitative PCR (qPCR) reactions were set up with SYBR Green (A25742, Applied Biosystems) and a final primer concentration 200 nM. qPCR was run with standard temperature settings on a Roche Lightcycler 480 instrument at the ISU Genome Technology Facility.

### Histology and immunolocalization

For histological analysis tumor tissue was fixed in 10% formalin, paraffin-embedded, microtome sections (6 µm) stained with Hematoxylin 7211 Richard-Allan Scientific (Thermo Fisher Scientific) and 3% Eosin Y (152880250, Argos Organics). For immunolocalization, tumor tissue and larval heads were fixed in 4% paraformaldehyde, embedded in Tissue-Tek OCT 4583 (Thermo Fisher Scientific) and sectioned at 16-20 µm. Cryosections were labeled with the following antibodies: rabbit polyclonal anti-phosphohistone H3 (9701, Cell Signaling Technology) at 1:1000; rabbit polyclonal anti-SOX2 (AB5603, EMD Millipore) at 1:200; rabbit polyclonal anti-SOX10 (GTX128374, GeneTex) at 1:200; rabbit polyclonal anti-OLIG2 (18953, IBL America) at 1:200; mouse monoclonal anti-HuC/HuD (A-21271, Invitrogen) at 1:300; rabbit polyclonal Living Colors DsRed (632496, Clonetech) at 1:200; rabbit anti-activated human caspase 3 (559565, BD Pharmingen) at 1:500. Alexa Fluor 488 (A-11008, Invitrogen)- and Alexa Fluor 594 (A-11005, Invitrogen)-conjugated secondary antibodies were used at a dilution of 1:500. Sections were stained with DAPI and mounted in Fluoro-Gel II containing DAPI (17985-50, Electron Microscopy Sciences). Immunolabeled sections were imaged on a Zeiss LSM700 laser scanning confocal microscope with 10×, 20×, 40× C-apochromat and 63× plan-apochromat objectives. Images and figures were assembled in Adobe Photoshop.

### Isolation of *Tol2<ubi:DsRed2>* transgenic line

The *Tol2<ubi:DsRed2>* transgene was assembled in the mini-*pTol2* vector (Balciunas et al., 2006) by cloning in the zebrafish *ubiquitin* promoter (Mosimann et al., 2011) followed by the *DsRed2* cDNA (Clonetech) and the zebrafish *β*-*actin* 3′UTR (Dr Darius Balciunas, Temple University) (McGrail et al., 2011). One microgram of linearized *pT3TS-Tol2* transposase plasmid (Balciunas et al., 2006) was used for *in vitro* synthesis of *Tol2* transposase-capped, polyadenylated mRNA using the T3 mMessage mMachine High Yield Capped RNA transcription kit (AM1348, Ambion). mRNA was precipitated with LiCl and resuspended in RNase, DNase-free molecular-grade water. The *Tol2<ubi:DsRed2>* transgenic line was isolated by co-injection of 25 pg *Tol2* vector plus 100 pg *Tol2* mRNA into one-cell-stage zebrafish embryos. Two founder fish were screened for germline transmission of ubiquitously expressed DsRed2. The *Tg(Tol2<ubi:DsRed2>)^is56^* line was established from a single F1 adult.

### Blastula cell transplants to create genetic mosaic zebrafish embryos

Transplant of cells from blastula-stage embryos was performed as described previously (Wang et al., 2010b); 30-50 cells from blastula stage embryos from adult *rb1Δ7/+* crossed to *rb1Δ7i/+*; *Tg(Tol2<ubi:DsRed2>)^is56^* were transplanted into blastula-stage *casper* host embryos, and the remaining donor blastula tissue was used for genotyping. Host embryos were screened for DsRed2-positive fluorescence at 24 and 48 hours postfertilization (hpf). Three host embryos containing DsRed2-expressing transplanted cells from three individual donor embryos of wild-type +/+ or homozygous mutant *rb1Δ7/Δ7* genotype were aged to 5 dpf. Larval heads were dissected, fixed, embedded, sectioned and immunolabeled for confocal imaging as described above.

### CRISPR-Cas9 guide RNAs and isolation of loss of function *rbbp4* and *hdac1* mutations

The following CRISPR guide RNAs (gRNAs) were used for somatic targeting of zebrafish: *rb1*, *rbbp4 and hdac1. rbbp4* and *hdac1* gRNAs were also used to isolate the stable germline 4 bp frameshift mutations *rbbp4Δ4-is60* and *hdac1Δ4-is70*. The *rb1* exon 2 CRISPR site 5′-GGAGAGGGAGATCAGATCGA-3′ contains a *Cla*I restriction enzyme site overlapping the Cas9 cut site upstream of the protospacer adjacent motif (PAM). The *rbbp4* exon 2 CRISPR site 5′-GATGACCCACGCCCTTGAG-3′ has a *Sml*I restriction enzyme site overlapping the Cas9 cut site. For *hdac1*, the exon 5 CRISPR site 5′-GACAGACAGTGCCATTAAC-3′ was used for targeting. CRISPR gRNAs were prepared by the cloning-free single-guide RNA synthesis method (Varshney et al., 2015) using Ambion T7 MEGAscript Kit (AM1334M, Ambion) and purified with the Qiagen miRNeasy Kit (217004, Qiagen). *Cas9* mRNA was synthesized *in vitro* using the mMessage mMachine T3 kit (AM1348, Life Technologies). Then, 5-10 μg of *pT3TS-nlsCas9nls* plasmid (46767, Addgene) was linearized with *Xba*I and purified with Qiaquick Spin kit (28104, Qiagen), 500 ng to 1 μg purified linear *pT3T3-nlsCas9nls* DNA was used as template for mRNA synthesis, and the capped *nls-Cas9-nls* mRNA was purified with the miRNeasy kit (Qiagen 217004).

Primers for amplicons to test for mutagenesis were as follows: *rb1* Forward 5′-TTTCCAGACACAAGGACAAGGATCC-3′, Reverse 5′-GCAGATATCAGAAGAAAGAGTACATTTGTCTT-3′; *rbbp4* Forward 5′-GCGTGATGACAGATCTCATATTGTTTTCCC-3′, Reverse 5′-CTGGTGACATCTGGCAACCACT-3′; *hdac1* Forward 5′-GCGGTGAAACTCAACAAACA-3′, Reverse 5′-GAATGGCCAGTACAATGTCG-3′.

To isolate stable germline mutations, 25 pg *rbbp4* or *hdac1* CRISPR guides were co-injected with 150 pg Cas9 mRNA into WIK embryos. Eight to ten targeted embryos were tested for mutagenesis by PCR genotyping, and sibling founders raised to adulthood. Adult founder fish were outcrossed to wild-type WIK fish and F1 embryos screened for inheritance of stable germline mutant alleles. Sibling F1 progeny were raised to adulthood and heterozygotes identified by genotyping of fin clip tissue. F2 families of heterozygous mutant fish were established by outcrossing to wild-type WIK.

### CRISPR somatic targeting and larval brain live imaging

CRISPR gRNAs were co-injected with 150 pg Cas mRNA into one-cell *Tg(H2A.F/Z-GFP)* embryos. At 12 hpf, embryos were placed in (0.003%) 30 mg/l phenyl-thio-urea (PTU) to inhibit melanin synthesis. Larvae at 5 dpf were mounted in 1.2% low-melt agarose containing 160 μg/ml tricaine methanesulfonate ventral side down on coverslip-bottom 35-mm petri dishes. The dish was filled with embryo water (Westerfield, 1995) containing tricaine and PTU. Live larvae were imaged on a Zeiss LSM700 laser scanning confocal microscope using a 40× W-N Achroplan water immersion objective. Z series containing 11 sections were captured beginning ∼30 μm below the dorsal side of the optic tectum and descending ventrally at 3-μm intervals. Three control uninjected *Tg(H2A.F/Z-GFP)* larvae and three CRISPR gRNA-injected *Tg(H2A.F/Z-GFP)* larvae for each gene were imaged. After imaging, larvae were digested in 50 mM NaOH and genotyped by PCR. Mitotic figures in each section were counted in ImageJ. Mitotic figure quantification was plotted and the significance calculated using a two-tailed unpaired Student's *t*-test in GraphPad Prism 7.0 (GraphPad Software).

## Supplementary Material

Supplementary information

First Person interview
