# Supplementary material for: Epigenetic regulators Rbbp4 and Hdac1 are overexpressed in a zebrafish model of RB1 embryonal brain tumor, and are required for neural progenitor survival and proliferation
Source: Dis Model Mech. 2018 Jun 15;11(6):dmm034124. doi: 10.1242/dmm.034124 (PMC6031359; doi:10.1242/dmm.034124)
Supplement: Supplementary information [file dmm-11-034124-s1.pdf]

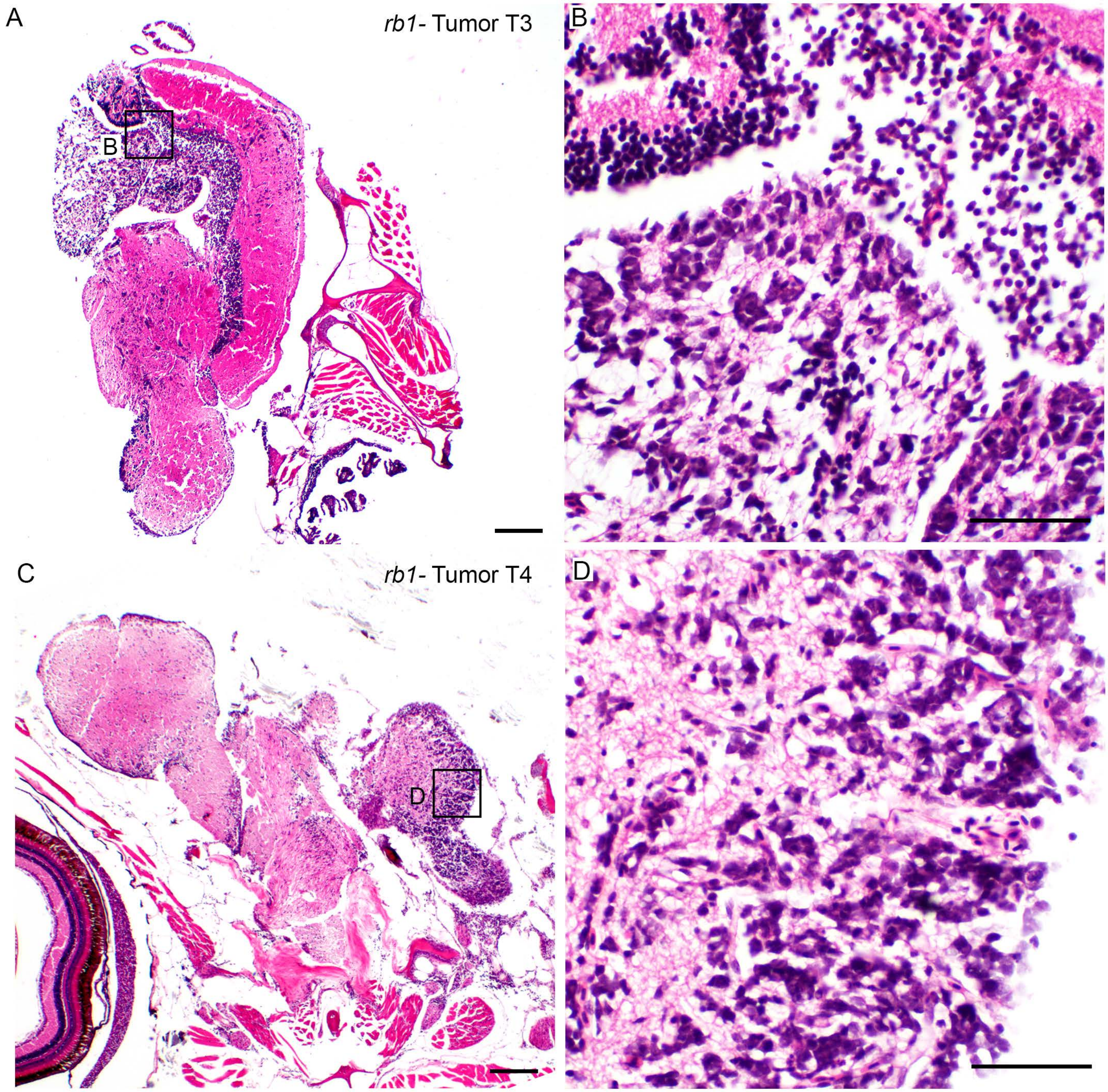

Figure S1. **Histological analysis of neoplastic populations in TALEN induced *rb1*-brain tumor T3 and T4 is consistent with primitive neuroectodermal-like tumor**. H&E stained sections of tissue remaining after dissection of tumors T3 (**A, B**) and T4 (**C, D**) used for transcriptome analysis. Sections exhibit an unencapsulated, multifocally infiltrative neoplastic population within the neuropil. Neoplastic cells are small with deeply basophilic, round to oval to wedge-shaped nuclei, densely clumped chromatin, and scant amounts of cytoplasm. Scale bars A, C 200μm; B, D 50 μm.

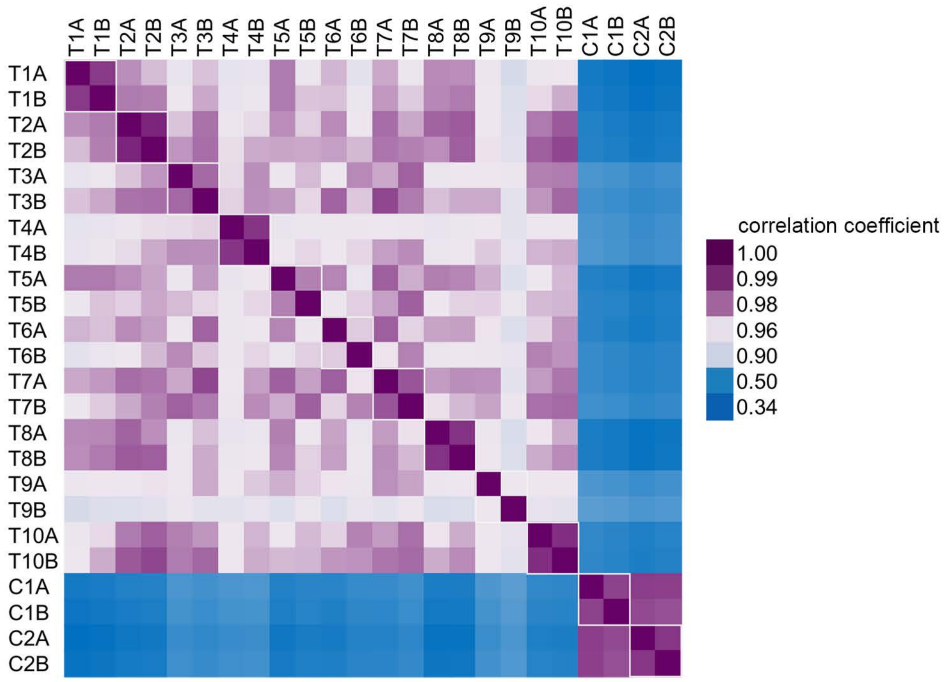

Figure S2. Correlation coefficients for RNA-Seq libraries from 10 *rb1*-brain tumor and two pools of 3 wildtype adult brains show high correlation between technical and biological replicates.

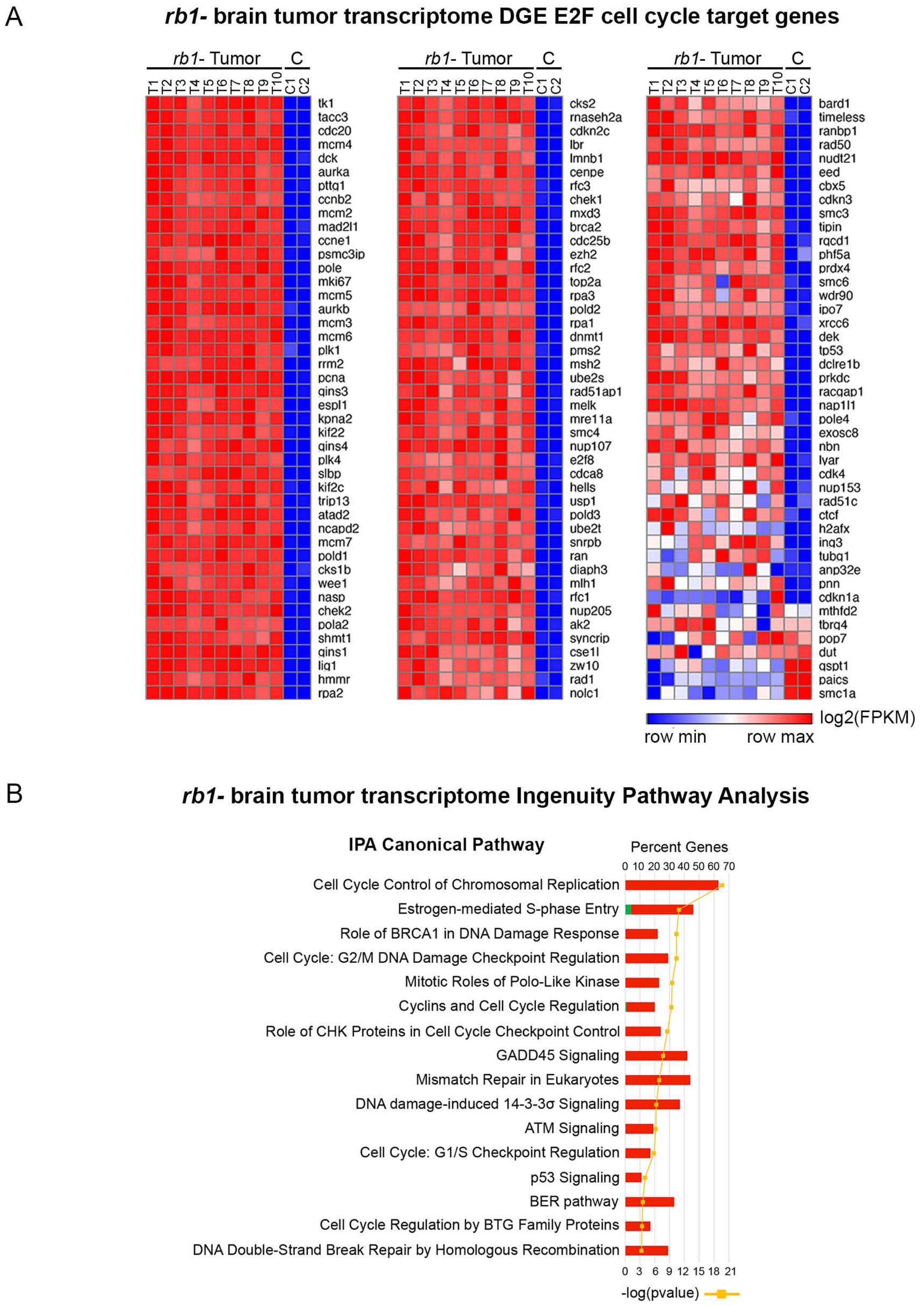

Figure S3. **Differentially expressed E2F target genes and Ingenuity Pathway Analysis of zebrafish *rb1*-brain tumor transcriptome. A** Heat map of differentially expressed E2F cell cycle target genes in *rb1*-tumor transcriptome (*rb1*-Tumor, T1-T10) compared to normal adult brain (C, C1-C2). **B** Ingenuity Pathway Analysis Canonical Pathways represented in the *rb1*-tumor transcriptome.

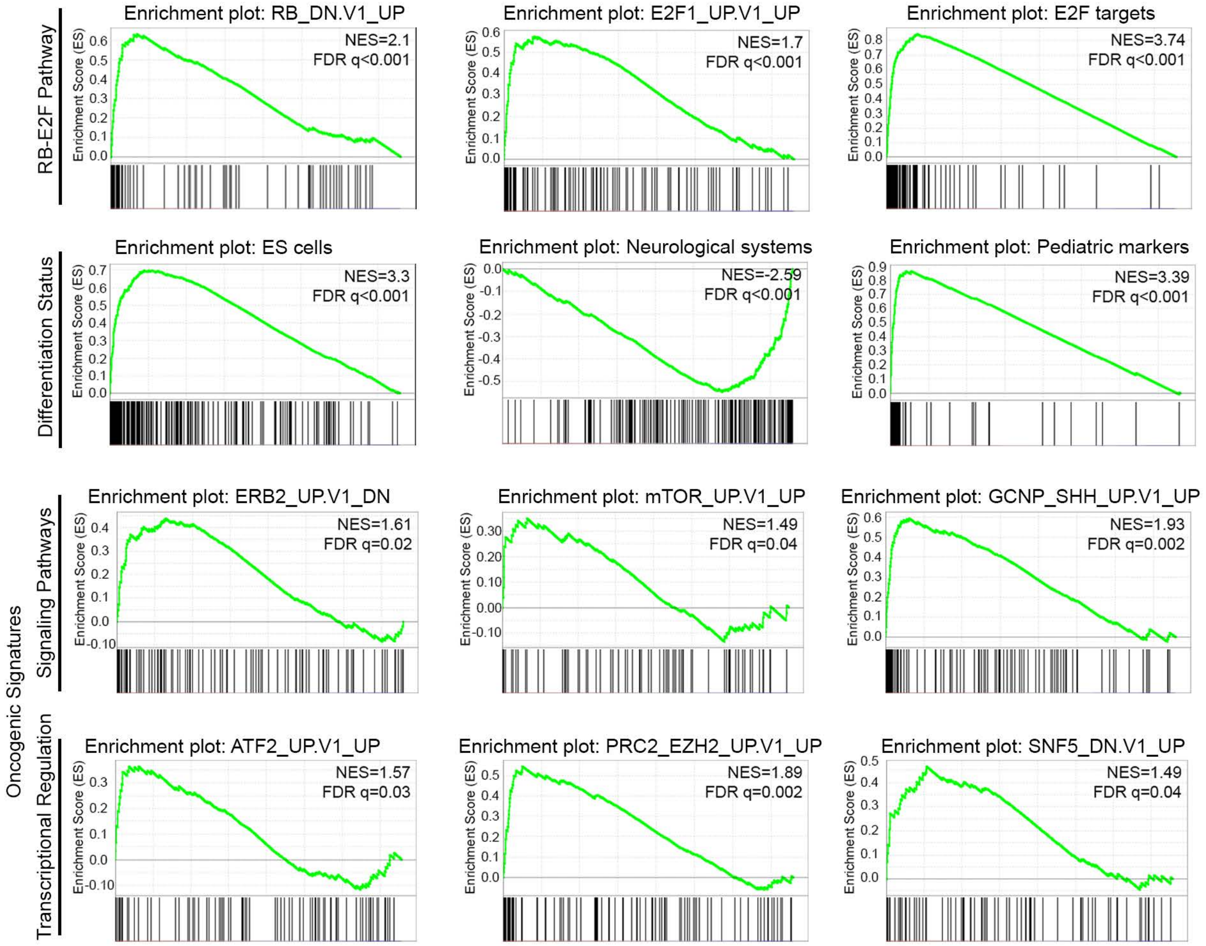

Figure S4. **Gene Set Enrichment Analysis of the *rb1*-tumor transcriptome**. GSEA shows positive correlation with RB-E2F, ES cell, pediatric tumor marker, ERB2, mTOR, SHH, ATF2, PRC2 and SNF5 pathways, and negative correlation with neurological systems.

Disease Models & Mechanisms • Supplementary information

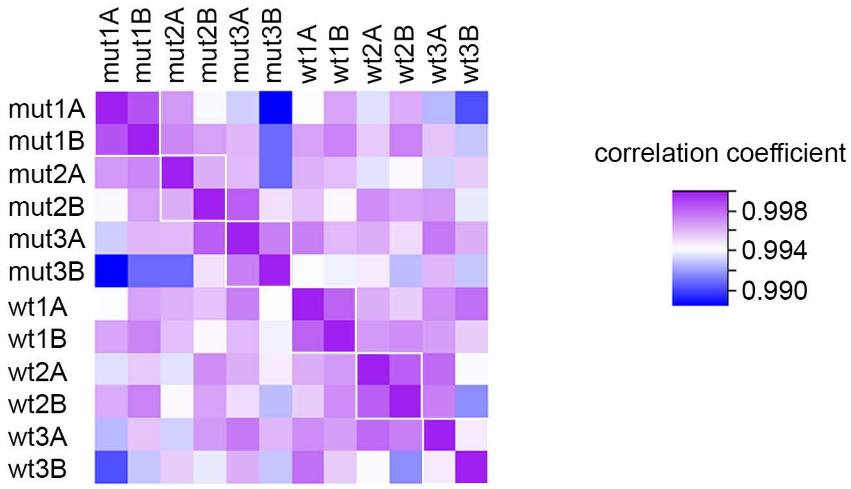

Figure S5. Correlation coefficients for RNA-Seq libraries from 3 *rb1Δ7/Δ7* and 3 +/+ 5 dpf larva show high correlation between technical and biological replicates.

Disease Models & Mechanisms • Supplementary information

A

***rb1*$^{\Delta 7/\Delta 7}$ mutant transcriptome E2F cell cycle target genes**

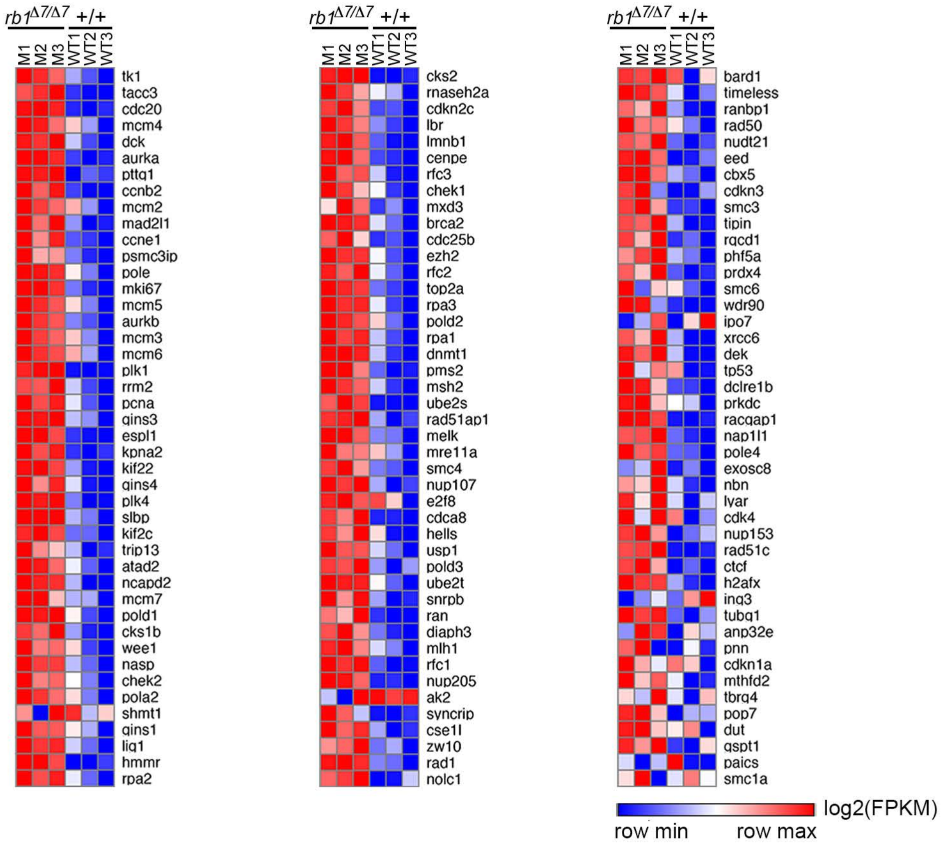

B

***rb1*$^{\Delta 7/\Delta 7}$ mutant transcriptome Ingenuity Pathway Analysis**

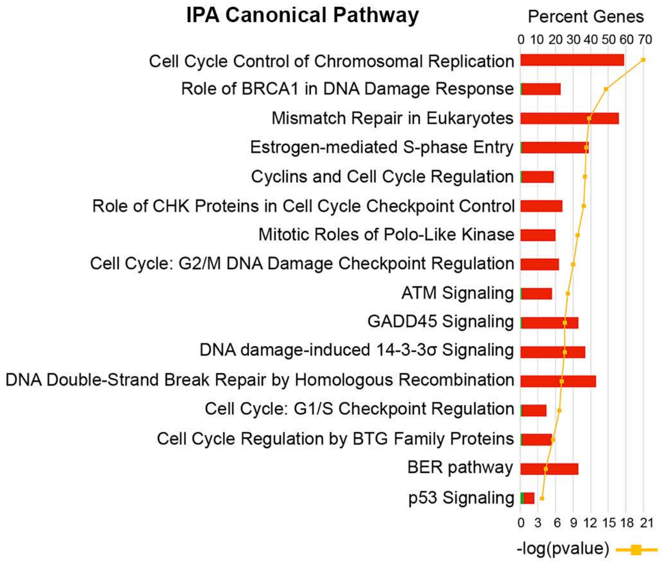

Figure S6. Differentially expressed E2F target genes and Ingenuity Pathway Analysis of zebrafish *rb1Δ7/Δ7* homozygous mutant 5pdf transcriptome. A Heat map of differentially expressed E2F cell cycle target genes in *rb1Δ7/Δ7* mutant transcriptome (M1-M3) compared to +/+ wildtype siblings (WT1-3). **B** Ingenuity Pathway Analysis Canonical Pathways represented in the *rb1Δ7/Δ7* mutant transcriptome.

Disease Models & Mechanisms • Supplementary information

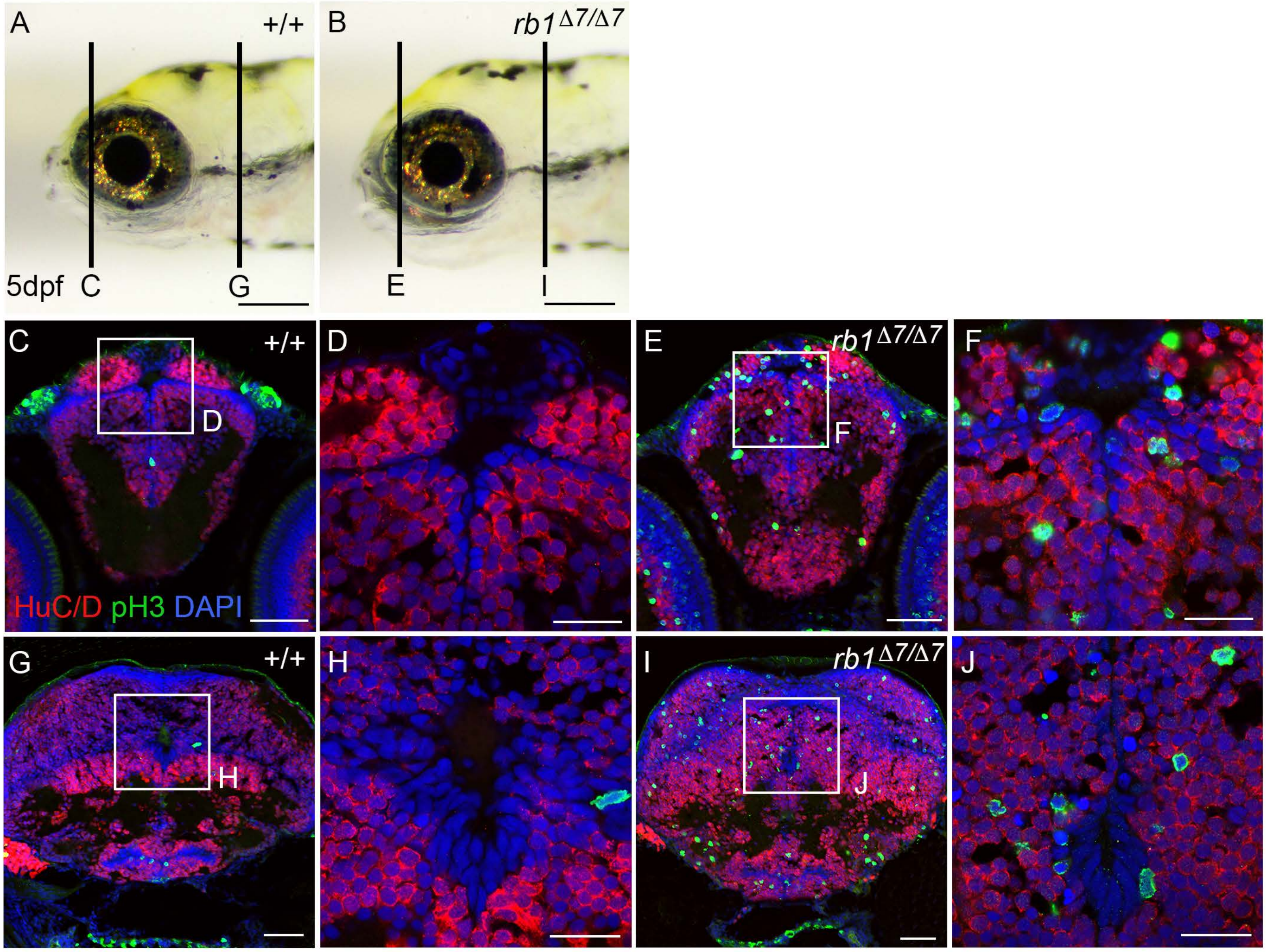

Figure S7. **Ectopic M-phase phosphohistone-H3 positive cells in the telencephalon/forebrain and hindbrain of *rb1Δ7/Δ7* mutant 5 dpf larva. A** wildtype +/+ and **B** *rb1Δ7/Δ7* mutant 5 dpf gross morphology. **C-J** Cryosections were labeled with antibodies to the neuronal marker HuC/D (red) and mitotic M-phase marker phosphohistone-H3 (green). Sections through the telencephalon (**C-F**) and hindbrain (**G-J**) show that in wild type mitotic cells are absent or rare, but in the *rb1Δ7/Δ7* mutant many ectopic phosphohistone-H3 positive cells are scattered throughout the region containing HuC/D-positive cell bodies of neurons.

Disease Models & Mechanisms • Supplementary information

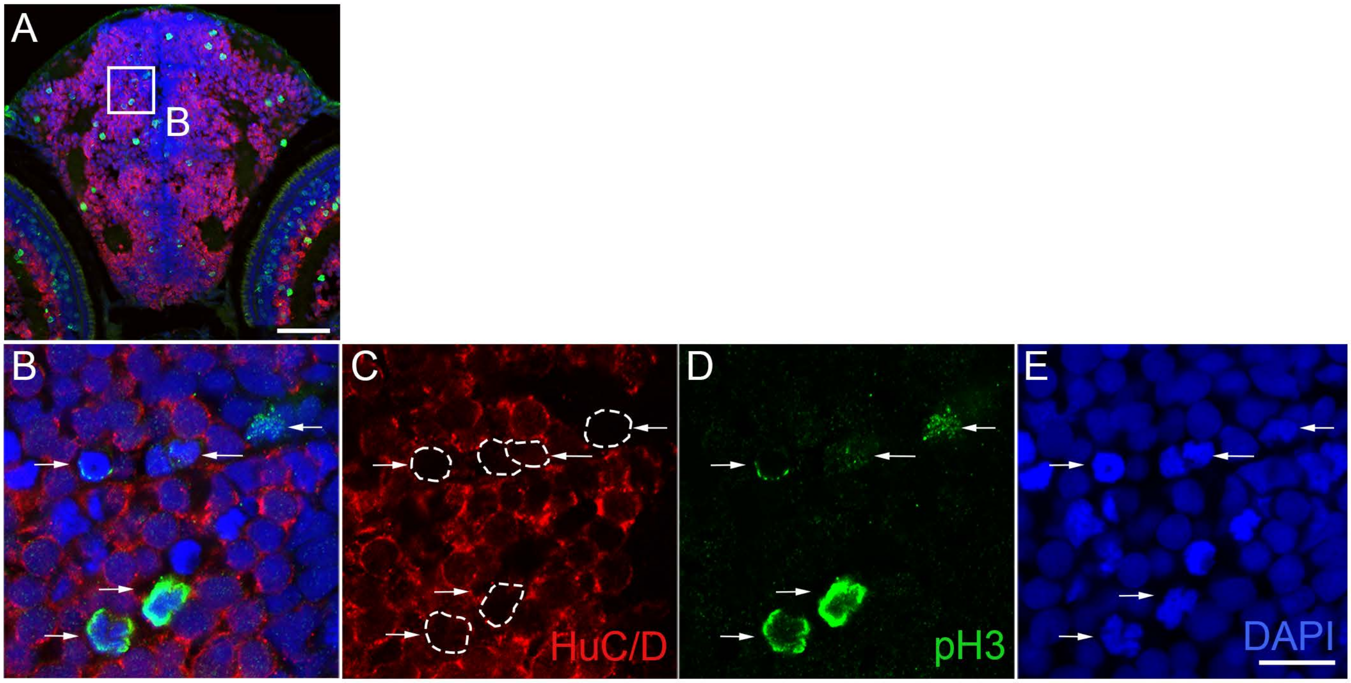

Figure S8. ***rb1Δ7/Δ7* mutant cells in M-phase lack expression of the neuronal differentiation marker HuC/D. A, B** High magnification image of phosphohistone-H3 positive nuclei (**B**, arrows) and HuC/D-positive cells in the tectum of a 5 dpf *rb1Δ7/Δ7* mutant brain. No overlap is detected between cells expressing HuC/D (**C**, red) and phosphohistone-H3 (**D**, green, arrows, dashed outlines). The chromatin in the phosphohistone-H3 positive cells appears highly condensed (**E,** arrows), consistent with cells in prophase/prometaphase of the cell cycle.

Disease Models & Mechanisms • Supplementary information

A *rb1* genotype of donor blastula embryos used for cell transplants to create genetic mosaic animals presented in Figure 3.

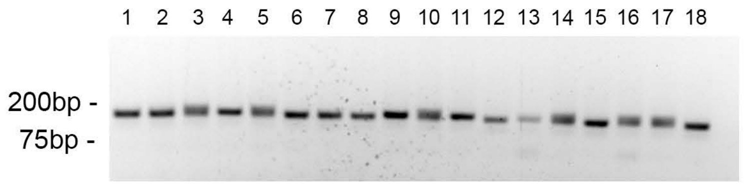

genotype	individual donor embryo	donor transplanted cells imaged in Figure 3
+/+	4, 9, 11, 13	13 (Figure 3 A, B, E, F)
rb1$^{\Delta7/+}$	3, 5, 10, 14, 16, 17	
rb1$^{\Delta7/\Delta7}$	1, 2, 6, 7, 8, 12, 15, 18	6 (Figure 3 C, D); 12 (Figure 3 G, H)

B *rb1* genotype of 5dpf *H2A.F/Z-GFP* larva imaged in time lapse Movies S2-S4.

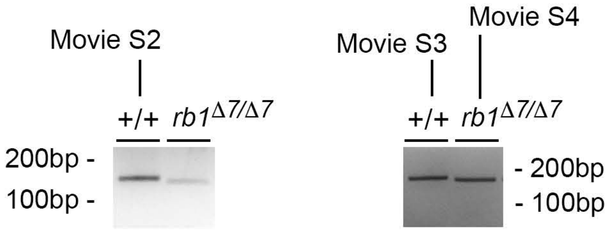

Figure S9. **Genotyping of blastula donor embryos used for cell transplants and 5 dpf larva used for time lapse imaging. A** PCR genotyping of remaining tissue in blastula stage donor embryos from *rb1Δ7/+* x *rb1Δ7/+*; *Tg(Tol2<ubi:DsRed2>)* after transplanting cells into host *casper* embryos. Genotype of individual embryos is shown below. Sections of host 5dfp larvae containing transplanted cells from +/+ embryo #13 and *rb1Δ7/rb1Δ7* embryos #6 and #12 are shown in Figure 3. **B** PCR genotyping of 5 dpf *+/+; Tg(H2A.F/Z-GFP)* and *rb1Δ7/Δ7; Tg(H2A.F/Z-GFP)* larva imaged in time lapse Movies S2-S4.

Table S1. Reads and quality control statistics from rb1-brain tumor and normal adult brain RNA-Seq libraries.

Table S1. RNASeq statistics for Control adult mid/hindbrain and *rb1-* defective Tumor transcriptome libraries

Sample	Yield (Mbases)	# Reads	% of >= Q30 Bases (PF)	Mean Quality Score (PF)
**Library Replicate A**				
T1A	718	14080104	94.03	37.46
T2A	754	14787466	94.11	37.47
T3A	815	15976577	93.93	37.42
T4A	770	15096943	94.11	37.47
T5A	822	16117019	94.02	37.46
T6A	865	16965370	94.04	37.46
T7A	843	16524639	94	37.44
T8A	817	16020463	93.95	37.43
T9A	628	12305355	94	37.45
T10A	668	13101532	93.82	37.39
C1A	722	14153617	93.91	37.42
C2A	721	14143448	94.08	37.47
**Library Replicate B**				
T1B	785	15387623	94.34	37.56
T2B	735	14412119	94.41	37.59
T3B	826	16188958	94.29	37.55
T4B	740	14514689	94.38	37.58
T5B	812	15931174	94.34	37.57
T6B	840	16466627	94.4	37.58
T7B	893	17516572	94.35	37.57
T8B	713	13981005	94.34	37.57
T9B	702	13767340	94.3	37.57
T10B	778	15247837	94.14	37.5
C1B	762	14940910	94.25	37.54
C2B	773	15158180	94.38	37.58

Library technical replicates A and B were sequenced by 50pb single read HiSeq Rapid Run in 2 separate lanes
T, RNASeq library from 10 tumors dissected from 10 individual adult fish
C, each control library 1 and 2 was generated from mid/hindbrain tissue dissected from 3 adults

Table S2. Gene read counts and FPKM for zebrafish *rb1*-brain tumor RNA-Seq libraries.

Click here to Download Table S2

Table S3. DGE zebrafish *rb1*-brain tumor RNA-Seq libraries.

Click here to Download Table S3

Table S4. Reads and quality control statistics from zebrafish homozygous mutant *rb1Δ7/Δ7* and wildtype +/+ 5 dpf larval head RNA-Seq libraries.

Table S4. RNASeq statistics for wildtype (wt) and *rb1Δ7/Δ7* mutant (mut) 5 dpf larval head transcriptome libraries

Sample	Yield (Mbases)	# Reads	% of >= Q30 Bases (PF)	Mean Quality Score (PF)
**Lane 1**				
mut1A	1588	31136059	96.55	38.31
mut2A	1605	31480228	96.5	38.29
mut3A	1476	28941078	96.55	38.31
wt1A	1538	30166621	96.56	38.31
wt2A	1383	27110928	96.52	38.3
wt3A	1430	30000242	96.57	38.32
**Lane 2**				
mut1B	1573	30850489	96.64	38.35
mut2B	1591	31191898	96.6	38.34
mut3B	1462	28659652	96.64	38.36
wt1B	1524	29882640	96.65	38.36
wt2B	1370	26854728	96.62	38.35
wt3B	1516	29722165	96.67	38.36

Libraries were sequenced by 50bp single read HiSeq Rapid Run in each of 2 separate lanes, A and B.
mut, 3 separate pools of 5 heads each from *rb1Δ7/Δ7* genotyped 5 dpf larvae.
wt, 3 separate pools of 5 heads each from +/+ genotyped sibling 5 dpf larvae.

Table S5. Gene read counts and FPKM for zebrafish *rb1Δ7/Δ7* mutant RNA-Seq libraries.

Click here to Download Table S5

Table S6. DGE zebrafish *rb1Δ7/Δ7* mutant RNA-Seq libraries.

Click here to Download Table S6

Disease Models & Mechanisms • Supplementary information

Table S7. Quantification of phosphohistone-H3 positive cells in wildtype and rb1 Δ7/Δ7 mutant 5dpf larval midbrain and retina

	# of phosphohistone-H3 positive cells				
	wildtype +/+			*rb1* Δ7/Δ7	
individual 5dpf larvae	midbrain	retina	-	midbrain	retina
1	2	1		85	89
2	3	0		62	103
3	6	5		50	83
4	7	4		69	98
5	5	3		62	97
6	3	2		61	99
7	5	3		83	115
8	4	3		63	120
9	3	1		59	73
10	0	2		67	101
11	4	2		57	103
12	1	4		63	75
13	6	4		42	93
14	2	4		75	97
15	3	2		81	92
16	5	0		71	50

Table S8. Primer sequences used for quantitative RT-PCR

Gene	Forward Primer	Reverse Primer
ascl1b	GCTGTGCTGTGGTGATAAACGT	CAAGCCTTGCCACTGCTCTT
hdac1	TACCACAGTGACGACTACATCAAGT	CAACAGATCCACCTGTTGAGAGC
metap1	GAACTGTGTGGAGAAAGAAGTCAACA	AAGGATGATCTGCATAGTCTGGTCT
olig2	AGCGAGATCTACGGCGGAG	TGGTGGAAGCAGAGGATGGT
rbbp4	CGCTTGAGTGGCCCAGTC	GCATCATCATTGGGCAGCTGA

Disease Models & Mechanisms • Supplementary information

Table S9. Quantification of activated caspase-3 positive cells in wt, *rbbp4/rbbp4* and *hdac1/hdac1* 2dpf larval midbrain and retina

	# of activated caspase-3 positive cells							
	wildtype +/+			*rbbp4* Δ4/Δ4			*hdac1* Δ4/Δ4	
individual 2dpf larvae	midbrain	retina	-	midbrain	retina	-	midbrain	retina
**1**	3	3		64	132		8	2
**2**	3	0		38	136		5	5
**3**	1	0		116	170		4	0

Table S10. Quantification of nuclei with condensed chromatin/mitotic figure in 5dfp larval tectum after *rb1, rbbp4, hdac1* CRISPR/Cas9 targeting

	# of nuclei with condensed chromatin/section														
	uninjected control larva				*rb1*-CRISPR targeted larva				*rbbp4*-CRISPR targeted larva				*hdac1*-CRISPR targeted larva		
confocal section	#1	#2	#3	-	#1	#2	#3	-	#2	#3	#4	-	#1	#2	#3
**1**	1	2	2		10	16	5		1	0	1*		1	0	0
**2**	1	3	0		6	12	10		1	0	8*		3	0	2
**3**	0	3	1		9	14	7		1	0	3*		2	0	1
**4**	1	2	0		8	14	9		1	0	2		0	0	1
**5**	0	2	1		11	16	9		0	0	1		2	0	1
**6**	1	3	0		11	17	10		1	0	1		4	0	3
**7**	3	3	0		14	21	3		2	0	1		3	2	0
**8**	2	1	1		20	15	7		0	0	0		0	1	0
**9**	3	1	2		11	12	4		0	0	0		0	0	0
**10**	1	1	4		15	11	6		1	0	0		1	0	0
**11**	1	2	4	-	18	16	6	-	1	1	3	-	1	0	0
**Total**	14	23	15		133	164	76		9	1	8		17	3	8

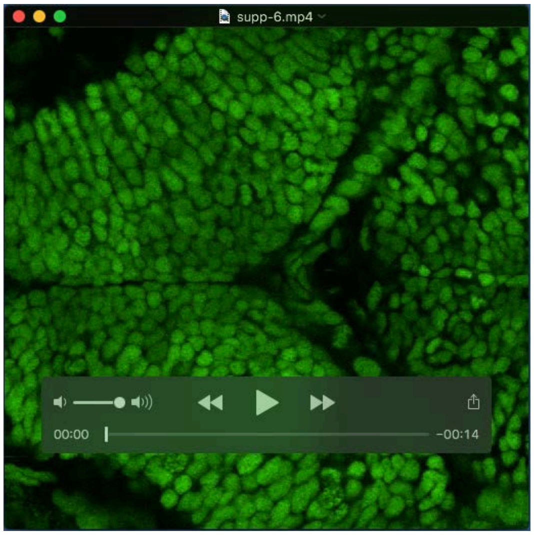

Movie S1. Live confocal time lapse movie of histone H2A.F/Z-GFP in dorsal optic tectum of a 3 dpf wild type larva. One-hour time lapse movie compressed to 10 frames/second.

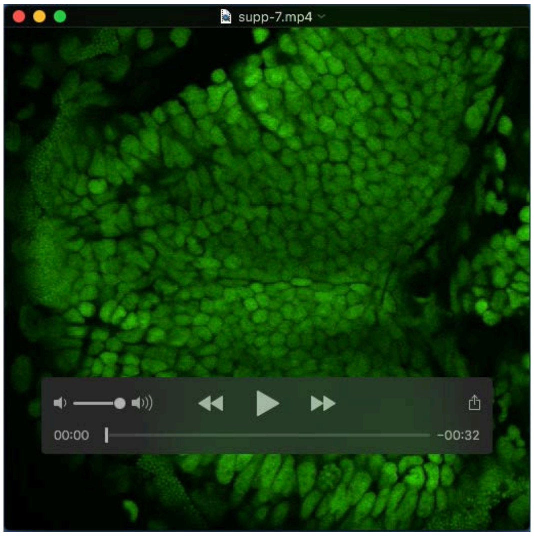

Movie S2. Live confocal time lapse movie of histone H2A.F/Z-GFP in dorsal optic tectum of a 5d pf wild type larva. Two-hour time lapse movie compressed to 10 frames/second.

Disease Models & Mechanisms • Supplementary information

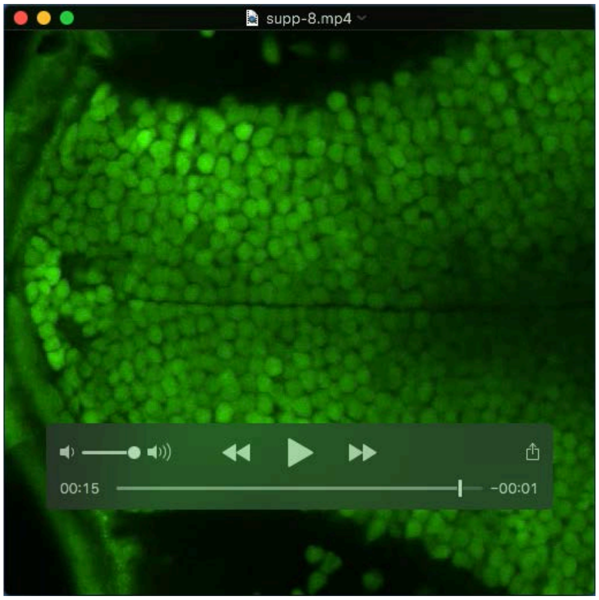

Movie S3. Live confocal time lapse movie of histone H2A.F/Z-GFP in ventral optic tectum of a 5 dpf wild type mutant larva. Two-hour time lapse movie compressed to 10 frames/second.

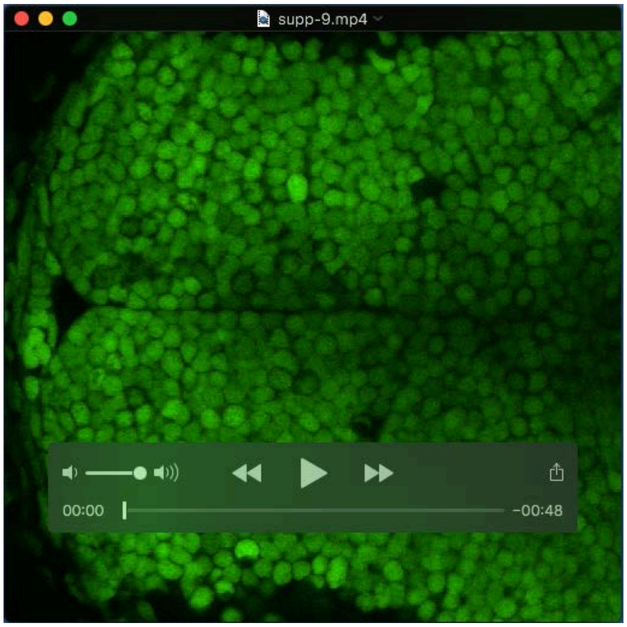

Movie S4. Live confocal time lapse movie of histone H2A.F/Z-GFP in ventral optic tectum of a 5 dpf *rb1Δ7/Δ7* mutant larva. Three-hour time lapse movie compressed to 10 frames/second.

Disease Models & Mechanisms • Supplementary information
