# Supplementary material for: Epigenetic regulators Rbbp4 and Hdac1 are overexpressed in a zebrafish model of RB1 embryonal brain tumor, and are required for neural progenitor survival and proliferation
Source: Dis Model Mech. 2018 Jun 15;11(6):dmm034124. doi: 10.1242/dmm.034124 (PMC6031359; doi:10.1242/dmm.034124)
Supplement: First Person interview [file dmm-11-034124-s2.pdf]

© 2018. Published by The Company of Biologists Ltd | Disease Models & Mechanisms (2018): doi:10.1242/dmm.034124: Supplementary information

FIRST PERSON

First person – Laura Schultz

First Person is a series of interviews with the first authors of a selection of papers published in Disease Models & Mechanisms, helping early-career researchers promote themselves alongside their papers. Laura Schultz is first author on 'Epigenetic regulators Rbbp4 and Hdac1 are overexpressed in a zebrafish model of RB1 embryonal brain tumor, and are required for neural progenitor survival and proliferation', published in DMM. Laura conducted the research in this article while a graduate research assistant in the lab of Maura McGrail at Iowa State University, Ames, USA, working on characterization of the epigenetic mechanisms driving neurogenesis and transformation in brain oncogenesis in the zebrafish. She is now a postdoctoral fellow at the Center for Individualized Medicine at the Mayo Clinic, Rochester, USA.

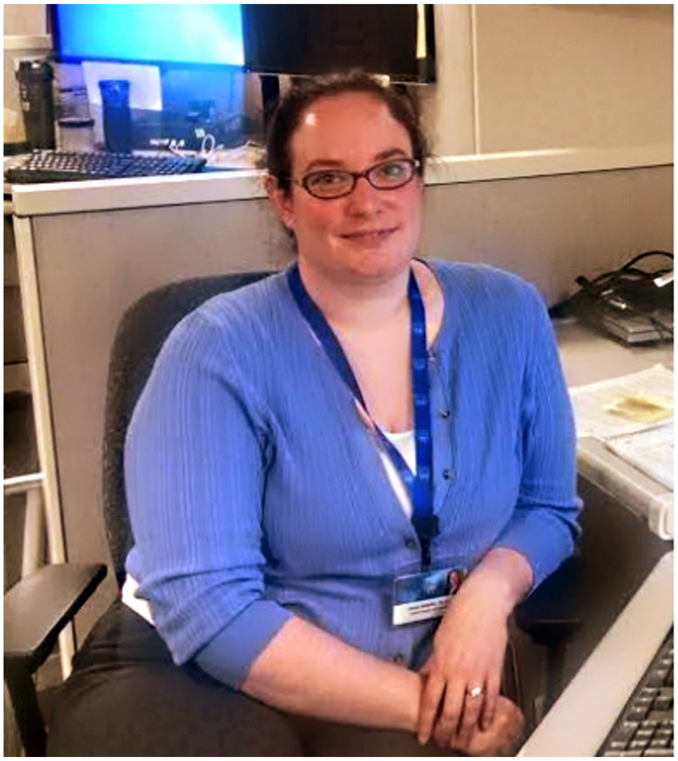

Laura Schultz

How would you explain the main findings of your paper to non-scientific family and friends?

Pediatric brain cancers can arise after mutation of one gene, a tumor suppressor gene called *retinoblastoma 1*. These tumors rarely contain other known cancer mutations, suggesting that chemical changes to the genome beyond DNA mutation – the epigenome – contribute to tumor growth. In our zebrafish model of *retinoblastoma 1* pediatric brain cancer we found high levels of genes that regulate the epigenome. To understand how these epigenetic regulators might contribute to brain tumor growth, we genetically disrupted their activity and examined how this affected brain development in living zebrafish embryos. We found that the genes are necessary for brain cell division and survival. In the future, we might be able to cause brain cancer cell death and block tumor growth by switching off these epigenetic regulators in *retinoblastoma 1* brain tumors.

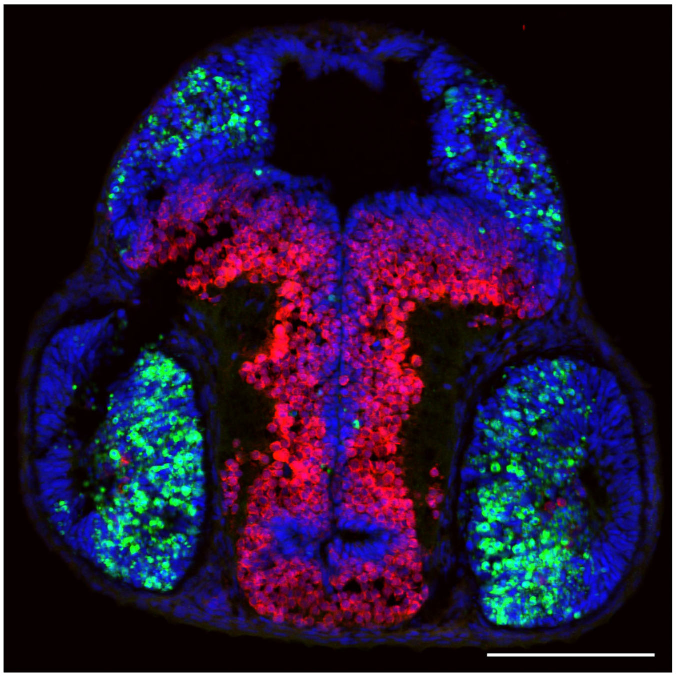

Immunolabeling of mutant zebrafish embryo midbrain at 2 days post-fertilization with neuronal marker HuC/D (red), apoptosis marker caspase-3 (green) and nuclear marker DAPI (blue) reveals extensive apoptosis in the neurogenic regions of the brain and retina.

What are the potential implications of these results for your field of research?

Epigenetic regulation of cell transformation and tumorigenesis is currently of great interest. Understanding how epigenetic mechanisms contribute to the complex nuclear changes occurring during oncogenesis is crucial for developing new strategies to limit tumor growth. The identification of chemical inhibitors that block the activity of epigenetic regulators is an important area for therapy development.

What are the main advantages and drawbacks of the model system you have used as it relates to the disease you are investigating?

The main advantage of zebrafish as a model system is the ease with which gene editing technology can be delivered to the externally fertilized single-cell embryo by simple injection. Highly efficient gene editing in zebrafish allows for rapid modeling of tumorigenesis and introduction of phenotypes in

Laura Schultz's contact details: 626 Science II, Iowa State University, Ames, IA 50010, USA.
E-mail: lauraeschultz@gmail.com

the targeted generation. The transparency of zebrafish embryos, combined with highly efficient CRISPR/Cas9 gene editing and live imaging, allowed us to rapidly assess the role of epigenetic regulators in neural cell proliferation and survival within five days of development. Normally, these types of studies require 3-6 months to isolate and characterize germline mutations. The main drawback to using zebrafish as a system to model brain cancer is its evolutionary distance from humans and less complex brain structure. However, the fundamental cellular mechanisms governing neurogenesis and oncogenesis appear to be conserved.

"Highly efficient gene editing in zebrafish allows for rapid modeling of tumorigenesis and introduction of phenotypes in the targeted generation."

What has surprised you the most while conducting your research?

After spending months analyzing fixed sections with two-dimensional imaging we began three-dimensional live confocal imaging of the developing brain. I was amazed to see how much complexity and dynamic activity was captured with time-lapse imaging that was missed in fixed sections.

Describe what you think is the most significant challenge impacting your research at this time and how will this be addressed over the next 10 years?

The biggest challenge is finding support for research in non-mammalian model systems. It's important that we continue to advertise the many advantages of a model system like zebrafish.

What changes do you think could improve the professional lives of early-career scientists?

I think an improvement for early-career scientists would be introducing alternative career options beyond just academia or industry. It would also be beneficial to help early-career scientists see how the skills they gain doing basic research can be translated to careers in clinical or informatics-based applications.

What's next for you?

I've begun a postdoctoral fellowship at the Center for Individualized Medicine at the Mayo Clinic, Rochester, MN. I'm part of the Diagnostic Odyssey group, which uses genetic analysis of whole exome sequencing and functional studies to help diagnose and understand rare genetic disorders in humans.

Reference

Schultz, L. E., Haltom, J. A., Almeida, M. P., Wierson, W. A., Solin, S. L., Weiss, T. J., Helmer, J. A., Sandquist, E. J., Shive, H. R. and McGrail, M. (2018). Epigenetic regulators Rbbp4 and Hdac1 are overexpressed in a zebrafish model of RB1 embryonal brain tumor, and are required for neural progenitor survival and proliferation. *Dis. Model. Mech.* **11**: dmm034124.
